# Nitrogen Fixation at the Edges of Boron Nitride Nanomaterials: Synergy of Doping

**DOI:** 10.3389/fchem.2021.799903

**Published:** 2022-01-21

**Authors:** Venkata Surya Kumar Choutipalli, Karthikraja Esackraj, Venkatesan Subramanian

**Affiliations:** ^1^ Inorganic and Physical Chemistry Laboratory, CSIR-Central Leather Research Institute, Chennai, India; ^2^ Centre for High Computing, CSIR-Central Leather Research Institute, Chennai, India; ^3^ Academy of Scientific and Innovative Research (AcSIR), Ghaziabad, India

**Keywords:** nitrogen reduction, catalysis, small molecules activation, DFT, doping, boron nitride, edge

## Abstract

Synthesis of ammonia at ambient conditions is very demanding yet challenging to achieve due to the production of ammonia fuel, which is considered to be a future fuel for sustainable energy. In this context, computational studies on the catalytic activity of the edge sites of boron nitride nanomaterials for possible nitrogen reduction into ammonia have been investigated. Geometrical and electronic properties of zigzag and armchair B-open edges of BN sheet (B_OE_) models have been unraveled to substantiate their catalytic nature. Results reveal that B_OE_ sites exhibit very high potential determining steps (PDS) of 2.0 eV. Doping of carbon (C) at the nitrogen center, which is vicinal to the B_OE_ site reduces the PDS of the N_2_ reduction reaction (NRR) (to 1.18–1.33 eV) due to the regulation of charge distribution around the active B_OE_ site. Further, the NRR at the C doped at various edge sites of a boron nitride sheet (BNS) has also been studied in detail. Among the 12 new C-doped defective BNS models, 9 model catalysts are useful for nitrogen activation through either chemisorption or physisorption. Among these, **ZC**_**N**_, **AC**_**N**_, and **ZC**_**BV**_ models are efficient in catalyzing NRR with lower PDS of 0.86, 0.88, and 0.86 eV, respectively. The effect of carbon doping in tuning the potential requirements of NRR has been analyzed by comparing the relative stability of intermediates on the catalyst with and without carbon doping. Results reveal that C-doping destabilizes the intermediates compared to non-doped systems, thereby reducing the possibility of catalyst poisoning. However, their interactions with catalysts are good enough so that the NRR activity of the catalyst does not decrease due to C-doping.

## Introduction

A century ago, ammonia was the savior of the world in the form of fertilizer which grew crops faster when food crises arrived due to the growing population (Aber et al., 1989; Bogaard et al., 2013; Galloway et al., 2017). For the extensive use of ammonia, Haber and Bosch developed an industrial method (the Haber-Bosch (HB) process) to synthesize ammonia artificially (Smith, 2002). The HB process addressed the world’s ammonia problem, and about 40% of the global population now relies on this process for ammonia synthesis (Howard and Rees, 1996; Kitano et al., 2015). However, the HB process is responsible for 1–2% of global energy consumption and 3% of CO_2_ emission due to the combustion of fossil fuels to generate H_2_ as the source of ammonia (Alexandratos and Bruinsma, 2012; Glibert et al., 2014; van der Ham et al., 2014; Zheng et al., 2020). Hence, the HB process should be relooked at for its environmental concerns. On the other hand, today’s major concern is climate change due to emission of carbon or green house gases (GHG). Hence, carbon-free energy storage is one of the major targets for sustainable energy and environment (Spatzal et al., 2014; McEnaney et al., 2017; Guo et al., 2018). In this context, ammonia is believed to be a future fuel owing to its advantages (Chehade and Dincer, 2021). Ammonia contains 17.8% of H_2_ by weight and its energy density by volume is nearly double that of liquid hydrogen (Lan and Tao, 2014; Kobayashi et al., 2019). Its physicochemical properties are similar to propane. Hence, the storage/transport methods developed for propane could also be used for ammonia. Therefore, by capturing, storing, and shipping hydrogen for use in emission-free fuel cells and turbines, ammonia is very handy (Guo and Chen, 2017; Kobayashi et al., 2019). Direct combustion of ammonia in power plants and ship engines leads to the carbon-free emission of fuel waste (Hinokuma et al., 2015; Chakraborty et al., 2017). Green production of ammonia, where oxidation of water is the source of H_2_ production thereby reducting N_2_ to synthesize ammonia, is the one of the important alternatives for carbon-free emission (Ye et al., 2017; Rouwenhorst et al., 2019; Yapicioglu and Dincer, 2019; Rouwenhorst et al., 2020; Salmon and Bañares-Alcántara, 2021). One more alternative is the electrochemical conversion of N_2_ to ammonia. Hence, it is obvious to develop efficient and sustainable electrocatalysts for the nitrogen reduction reaction (NRR).

**Graphical Abstract:**
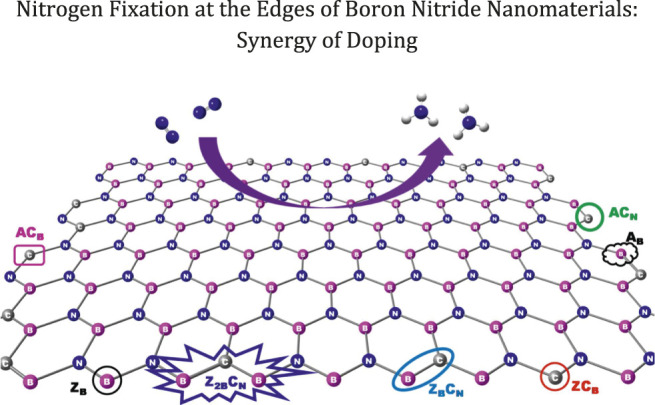


Two-dimensional (2D) materials are widely employed for various applications owing to their unique properties (Schwierz et al., 2015; Wang et al., 2016; Ashworth and Foster, 2018; Wang et al., 2018; Zhu et al., 2021). These materials exist as multi-layered materials with one-atom thick planes, which stack on top of each other (Mittal et al., 2015). Weak attractive van der Waals forces mainly govern the stabilization of these stacked layers. The surface parallel to the plane of 2D materials is called the basal plane, and the edge plane refers to the plane that is perpendicular to the surface. The basal plane surface shows atomic flatness and low defect density. On the other hand, the edge plane contains dangling bonds and defects (Velický et al., 2019). Some of the functional groups are seen at the edges due to abrupt lattice termination and their reactivity. Hence, the edge sites display special properties, *viz*., unsaturated coordination, accumulated charge density, and spin density (magnetic properties) (Fujii and Enoki, 2013; Zhang et al., 2013; Fujii et al., 2014; Bellunato et al., 2016). Previously, comparative studies on the reactivity of the edge and the graphene’s basal plane have been made (Randin and Yeager, 1972; Bowling et al., 1989; Rice and McCreery, 1989). Various experimental revelations on adsorption, electron transfer (ET), and capacitance demonstrated that the edge plane is electrochemically active whereas the basal plane either exhibits vanishingly low electrochemical activity or is completely inactive (Randin and Yeager, 1975; McDermott et al., 1992; Banks et al., 2005). Recent advances in electrochemical imaging and localized electrochemical measurements have clearly elucidated that the edges and defects are more reactive than the basal plane (Davies et al., 2005; Tan et al., 2012; Zhang et al., 2014; Zhong et al., 2014).

Atomic layer thin h-BN can also exhibit different properties due to the distinct structure of their edge states (Ooi et al., 2006; Golberg et al., 2010; Zeng et al., 2010). For example, doping of carbon at either the boron or nitrogen site of h-BN exhibits magnetism (Okada and Oshiyama, 2001; Li et al., 2009). It is well known from previous studies that boron-based catalysts have a high potential for the nitrogen reduction reaction (Zhao and Chen, 2017; Zhang et al., 2019). Therefore, it is natural to expect that these boron edges of h-BN may catalyze N_2_ reduction. Boron-based activation involves an “acceptance-donation” mechanism between B and N_2_, which is similar to that of transition metal-assisted N_2_ activation (Lv et al., 2019; Shi et al., 2019). Here, the binding between boron and hydrogen has been inhibited by an *sp* ^*3*^-hybridized boron atom, suppressing the HER in acidic conditions (Hering-Junghans, 2018; Yu et al., 2018; Liu et al., 2019). Similar structural features can be found in B-centers of BN nanomaterials. Previous studies have shown that edge states of 2D materials are highly reactive which are regulated by the edge centers (Velický and Toth, 2017; Momeni et al., 2020). The adjustable edge structures fine-tune these reactivities. These intrinsic features are responsible for making these edges highly efficient catalytic sites. Therefore, the role of edge sites in catalyzing NRR has been investigated systematically using density functional theory (DFT)-based methods. Both zigzag and armchair edges have been considered for evaluation. In addition, the effect of carbon doping at various edge sites has also been investigated. All the possible mechanistic pathways have been traced to establish the minimum energy reaction pathway for the nitrogen reduction reaction. The present computational exploration of catalytic activities of B-centers would definitely add value to the knowledge portfolio of this research topic and accelerate the development of NRR catalysts.

### Computational Details

A DFT-based Perdew-Burke-Ernzerhof (PBE) (Adamo and Barone, 1999) hybrid functional method was adopted with a 6-31G(d) (Hariharan and Pople, 1973; Hehre et al., 1986) basis set. The non-covalent interactions between the systems were modeled using Grimme’s dispersion-corrected (PBE-D3) functional theory (Grimme et al., 2010). Vibrational frequency analysis was carried out to verify whether the optimized geometries were in minima or maxima on the potential energy surface. The absence of imaginary frequency criteria was used to characterize the minimum energy geometry. As the calculations involve intermediates with open-shell configuration, spin-polarized (unrestricted) calculations were performed. Population analysis was carried out at the same level of theory to find the charge transfer at each elementary step of the reaction. All the quantum chemical calculations were performed with the G09 suite of the program (Frisch et al., 2009). Density of states (DOS) calculations were obtained at the GGA-PBE level as implemented in the DMol^3^ program (Delley, 2000) using the optimized geometries calculated at the PBE/6-31G(d) level of theory.

The feasibility of nitrogen adsorption on the catalyst was predicted by computing the interaction energy (E_ads_) using the following equation:

$$E_{ads}=\;E_{adsorbate+catalyst}-\left(E_{adsorbate}+\;E_{catalyst}\right)$$ (1)

Where E_adsorbate/catalyst_, E_adsorbate_, and E_catalyst_ are the total energies of the adsorbate−catalyst, the isolated adsorbate, and the defective BNS, respectively. According to this definition, a negative E_ads_ indicates exothermic adsorption.

The Computational Hydrogen Electrode (CHE) (Nørskov et al., 2004; Rossmeisl et al., 2005) model was applied for simulating the effect of the concerted transfer of protons and electrons to all the elementary steps of the reaction. The model involves the calculation using the formula as given in Eq. 2.

$$\frac{1}{2}G_{H_{2}}=G\left(H^{+}+E^{-}\right)$$ (2)

## Results and Discussion

### Geometries of Model Systems

**FIGURE 1:**
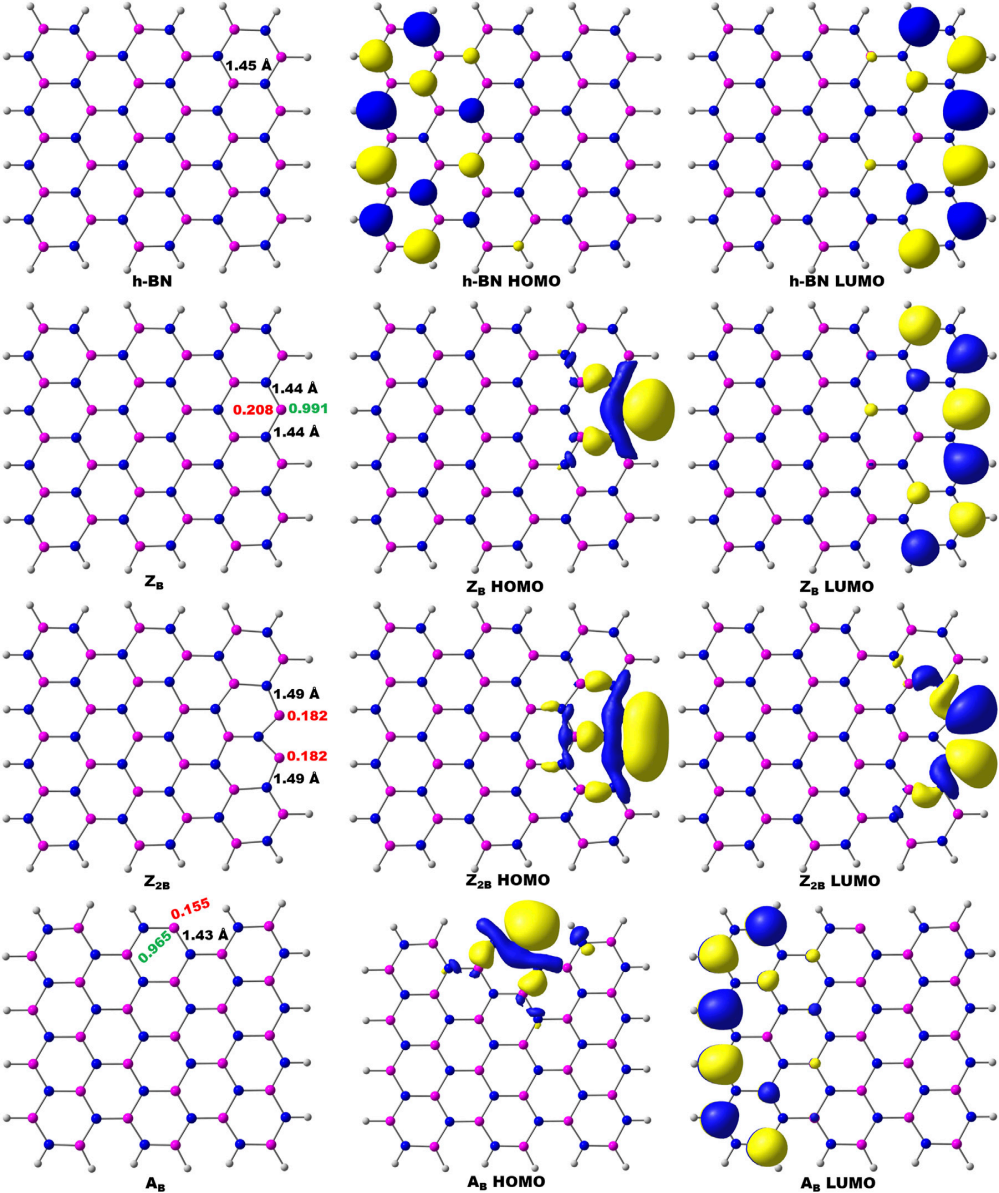
Optimized geometries of pristine and B-open edge defective BNS calculated at the PBE/6-31G(d) level of theory. Frontier molecular orbitals of the corresponding systems are given. Charge and spin densities on the active site are shown in red and green color, respectively. Color indication: Pink-B, Blue-N, and White-H.

Both zigzag and armchair boron nitride nanomaterials have been considered to assess the catalytic properties of their edge centers. The model systems chosen for this investigation are depicted in Figure 1. For brevity, the following nomenclature was used throughout the text. Zigzag and armchair models are denoted as **Z** and **A**. BN systems with one and two B-open edges (B_OE_) at the zigzag edge are called **Z**_**B**_ and **Z**_**2B**_, respectively. Similarly, the same at the armchair edge is referred to as **A**_**B**_. The symbols **C**_**B**_ and **C**_**N**_ represent carbon doping at boron and nitrogen sites, respectively. Similarly, **C**_**BV**_ and **C**_**NV**_ refer to carbon doping at boron-vacancy and nitrogen-vacancy, respectively. The notation, **N**_**x**_ **H**_**y**_ **H**_**z**_ (x = 1,2 and y, z = 0–6) represents different intermediates observed in the nitrogen reduction reaction.

**FIGURE 2:**
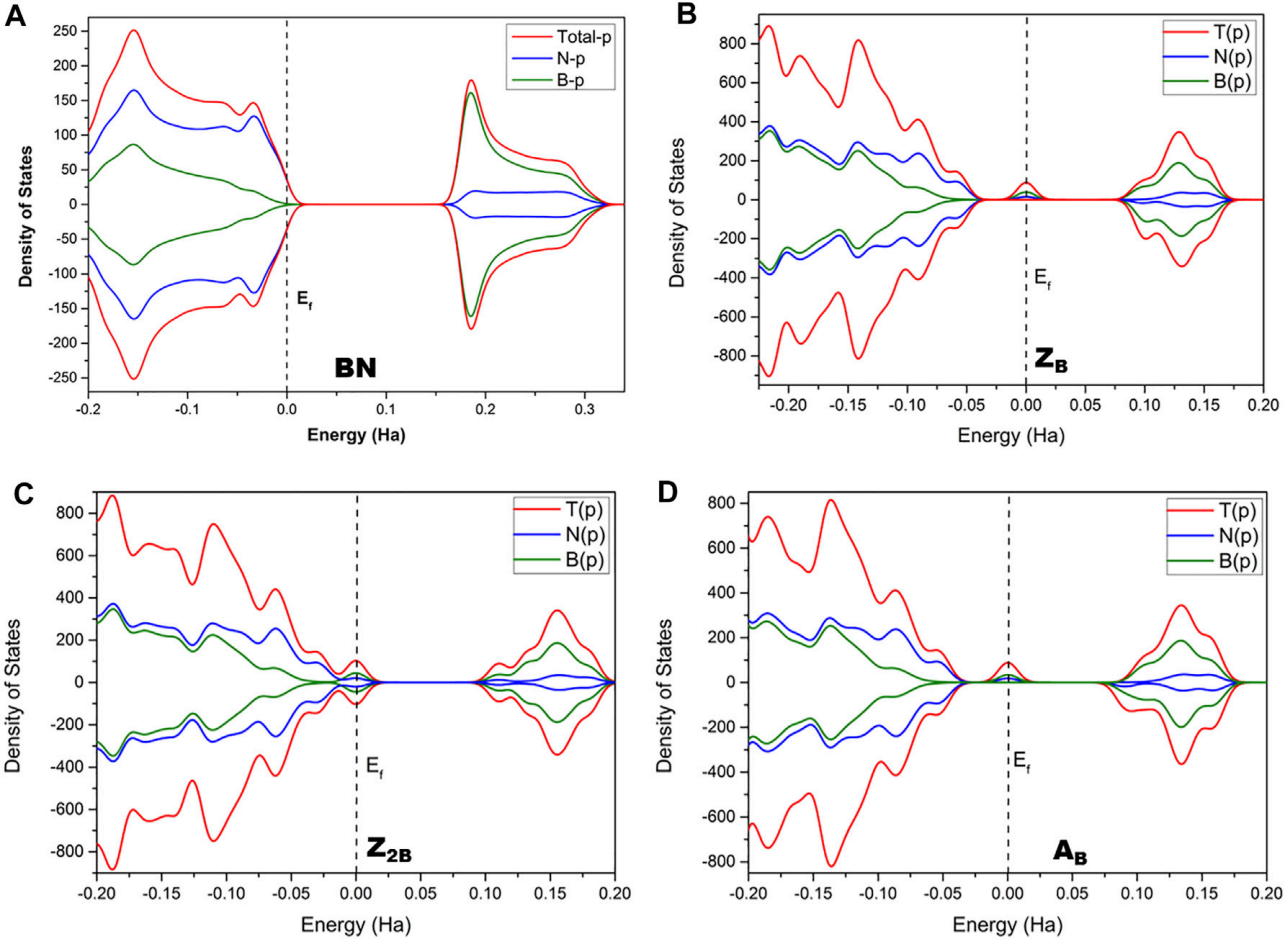
Calculated density of states (DOS) of **(A)** pristine h-BNS, **(B) Z**_**B**_, **(C) Z**_**2B**_, and **(D) A**_**B**_ defective BNS.

The conventional nitrogen reduction process is initiated with the activation of N_2_ by the surface of the models. This step is very important for the subsequent reduction steps to form ammonia. However, these steps are significantly affected by the electronic properties of the model catalysts. Hence, a prior understanding of the geometrical and electronic properties of designed models is important. In that context, it is necessary to compute the DOS of pristine BNS and BNS with defects such as zigzag open edge and armchair open edge. The calculated results are depicted in Figure 2. In addition, the possibility of formation in such defective systems is also a very important aspect to be considered for their experimental feasibility. Hence, defect formation energies of **Z**_**B**_, **Z**_**2B,**_ and **A**_**B**_ have been calculated as 4.17, 8.29, and 4.10 eV, respectively which are in the range of experimental limitations. Hence, these results indicate their experimental feasibility. The defect formation energies of all the defective systems that are considered in this study have been given in Table 1.

**TABLE 1:** Calculated cohesive energy (E_coh_) and defect formation energies (E_def_) of BN systems at the PBE/6-31G(d) level of theory.

S. No	Model	E_coh_ (eV)	E_def_ (eV)	S. No	Model	E_coh_ (eV)	E_def_ (eV)
1	**Z**_**B**_	−5.59	4.16	10	**ZC**_**N**_	−5.59	4.02
2	**Z**_**2B**_	−5.61	8.29	11	**ZC**_**N**_ **H**	−5.58	−0.47
3	**A**_**B**_	−5.67	4.10	12	**ZC**_**NV**_	−5.56	0.60
4	**Z**_**B**_ **C**_**N**_	−5.62	−1.70	13	**AC**_**B**_	−5.62	1.94
5	**Z**_**2B**_ **C**_**N**_	−5.64	−2.11	14	**AC**_**B**_ **H**	−5.58	−0.42
6	**A**_**B**_ **C**_**N**_	−5.62	−1.87	15	**AC**_**BV**_	−5.57	0.42
7	**ZC**_**B**_	−5.62	2.31	16	**AC**_**N**_	−5.59	4.01
8	**ZC**_**B**_ **H**	−5.59	−0.78	17	**AC**_**N**_ **H**	−5.57	−0.11
9	**ZC**_**BV**_	−5.57	0.79	18	**AC**_**NV**_	−5.57	0.28

It is well known that h-BN is a wide bandgap insulator, and experimental band gap values are in the range of 4.0–5.6 eV (Panchakarla et al., 2009; Golberg et al., 2010; Wu et al., 2013; Xue et al., 2013). It does not exhibit magnetism. The calculated value from this investigation is found to be 4.25 eV. It is already reported that PBE functional underestimates band gap values (Janesko, 2011; Hollins et al., 2012; Pari et al., 2016). Moreover, this value may arise from the effect of edge passivation of chosen model systems by hydrogen. However, our group has demonstrated the usefulness of truncated models as replicas for periodic 2D materials and catalysis of oxidation of alcohols into aldehydes (Vijaya Sundar and Subramanian, 2013; Jeyaraj et al., 2015). In the case of B_OE_, the partial wave appears at the Fermi level, indicating the conducting nature of defective BN systems. Thus, these systems are suitable for catalytic applications due to defective sites and associated effective electron transfer.

Further, charge densities on the active sites have also been analyzed in detail to understand the activity of B_OE_ sites. In pristine h-BN (passivated by hydrogen), the charge density on the boron atom of the boron edge is 0.26 e. The charge density for the B-open edge site is 0.20 e. It is noted from the results that the pristine BNS does not exhibit any spin density and is non-magnetic. However, the spin density on **Z**_**B**_ is 0.99, which is localized on a B_OE_ indicating its magnetic behavior. In similar lines, **Z**_**2B**_ exhibits a reduced positive charge (0.25 e) and increased spin density (0.97) on open B-sites. These inherent properties of defective systems render the open B-site of **Z**_**B**_ highly reactive when compared to the pristine BN.

### N_2_ Adsorption

**FIGURE 3:**
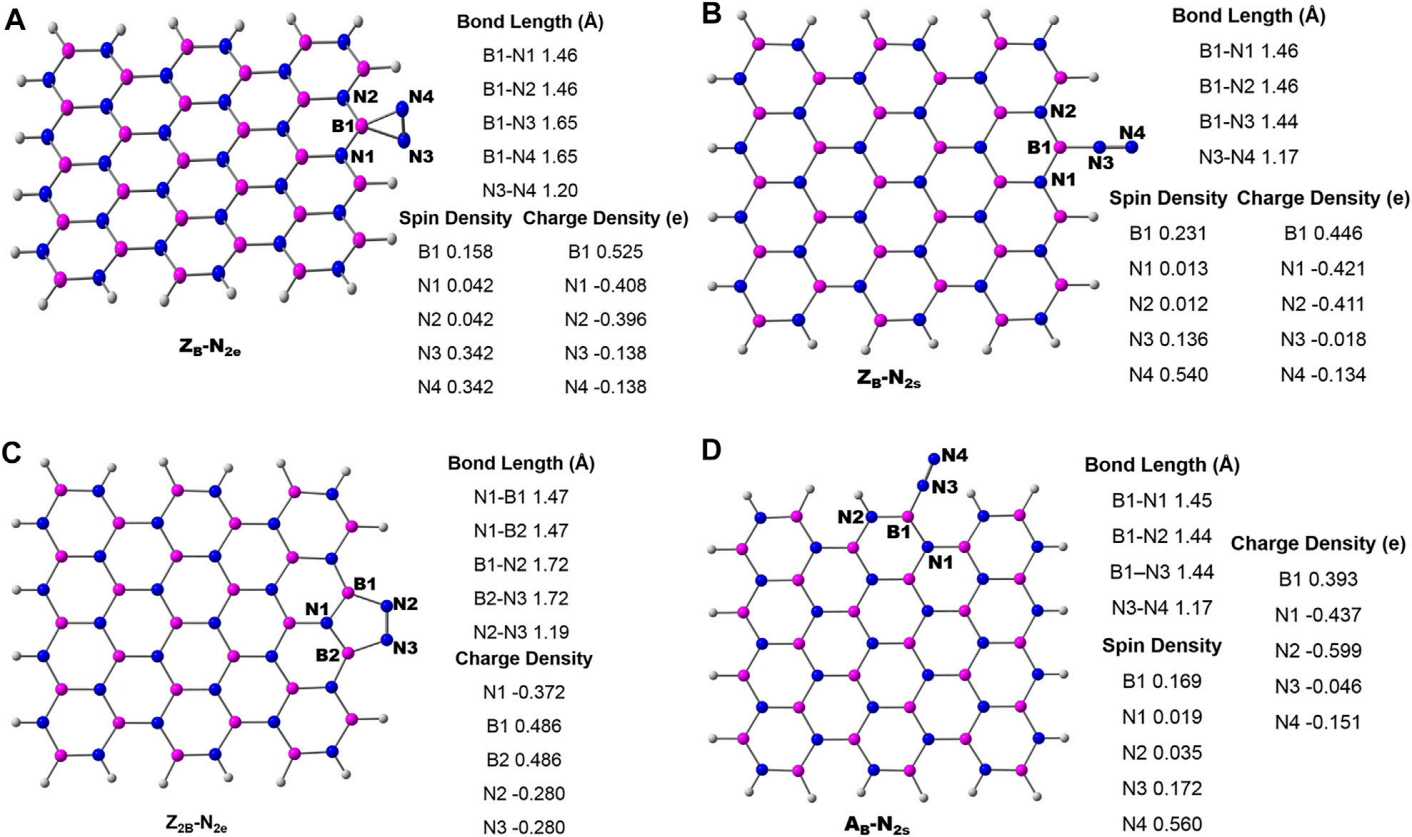
Optimized geometries of various possibilities of N_2_ adsorption on B-open sites of BN systems. **(A)** Side-on mode on **Z**_**B**_, **(B)** end-on mode on **Z**_**B**_, **(C)** side-on mode on **Z**_**2B**_, and **(D)** side-on mode on **A**_**B**_ calculated at the PBE/6-31G(d) level of theory. Color indication: Pink-B, Blue-N, and White-H.

The two possible modes of the adsorption pattern of N_2_ on the surface are end-on (Schrock) and side-on (enzymatic) modes. In the end-on adsorption mode, N_2_ binds to the active site through one of its nitrogen atoms (N_2s_). While in the side-on mode, N_2_ molecule binds parallel (*N_2e_) to the surface of BNS. The optimized geometries of N_2_ adsorption modes on B_OE_ are given in Figure 3. Both the modes are energetically possible for **Z**_**B**_ with the adsorption energies of -0.57 (end-on) and −0.16 eV (side-on). The corresponding adsorption elongates the N-N bond from 1.11 to 1.16 Å (end-on) and 1.11 to 1.20 Å (side-on). In this process, N_2_ gains 0.15 e from **Z**_**B**_ in the end-on mode. The same for the side-on mode is 0.28 e. The high charge transfer and concomitant elongation of the N-N bond imply the activation propensity of **Z**_**B**_ in enzymatic mode. Similarly, results from N_2_ activation by **Z**_**2B**_ reveal that the side-on mode dominates the end-on mode. In this mode, **Z**_**2B**_ exhibits high interaction energy of −2.39 eV with N_2_ leading to a charge transfer of 0.56 e from **Z**_**2B**_ to N_2_. This interaction is responsible for the appreciable lengthening of N-N from 1.11 to 1.28 Å due to the availability of more B-active sites for interaction.

An armchair B-open edge (A_B_) exhibits only the end-on mode of N_2_ adsorption (Figure 3D), despite many trials in different orientations. The adsorption energy of this mode is −0.32 eV with a charge transfer of 0.19 e from **A**_**B**_ to the N_2_ molecule. The N-N bond length increases from 1.11 to 1.17 Å. It is clear that the zigzag edge of BN nanomaterials can activate nitrogen. Specifically, the energetics of adsorption through the side-on mode on **Z**_**2B**_ indicates a chemisorption process. Considerable transfer of electron density from the BNS surface to N_2_ accompanies this activation along with appreciable lengthening of the N-N bond. It is pertinent to mention that the strong interaction between BNS and the substrate leads to catalyst poisoning due to difficulties in the desorption of products. The calculations show that the N-open edge cannot activate N_2_. Hence, the reduction of N_2_ using the B-open edge model has been taken for detailed elucidation.

### N_2_ Reduction at B-Open Site Edge BN Systems

**FIGURE 4:**
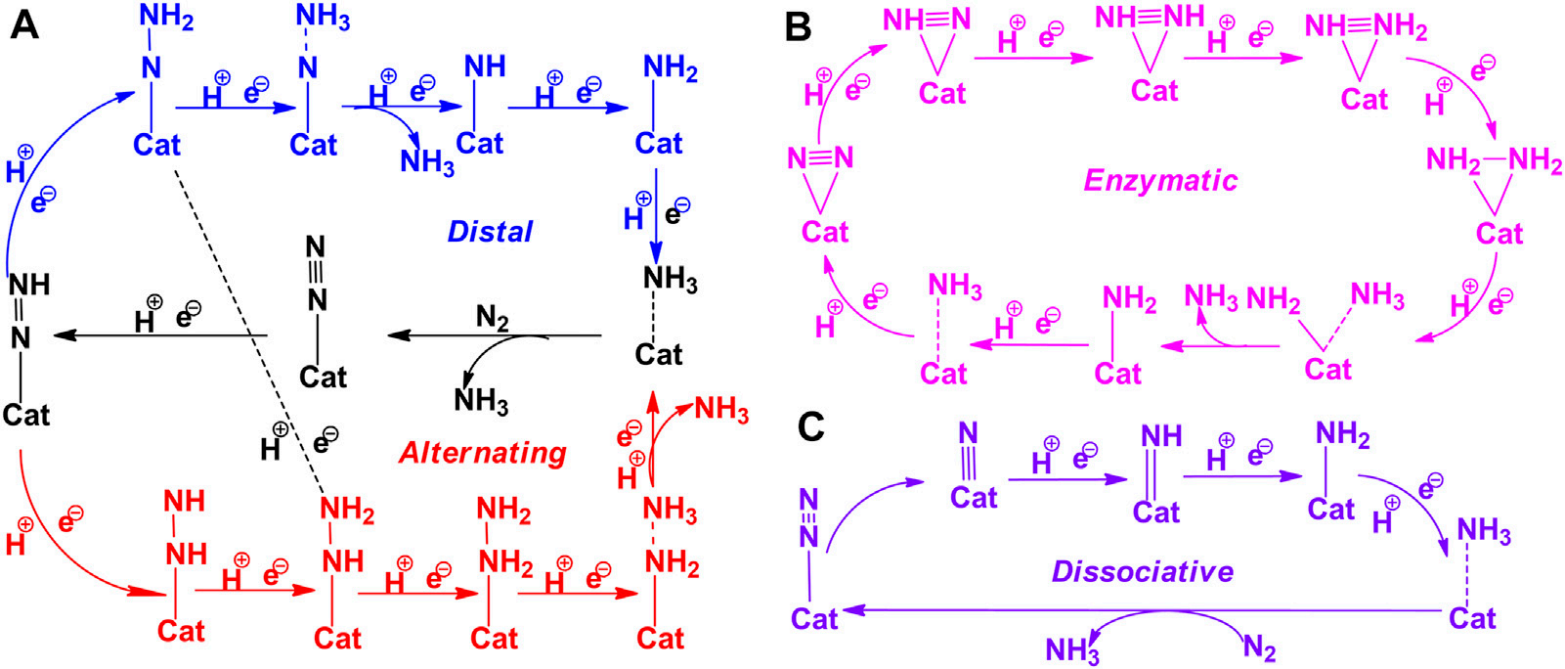
The schematic representation of nitrogen reduction reaction pathways. **(A)** Distal (blue color) and alternating (black color), **(B)** enzymatic, and **(C)** dissociative pathways.

**FIGURE 5:**
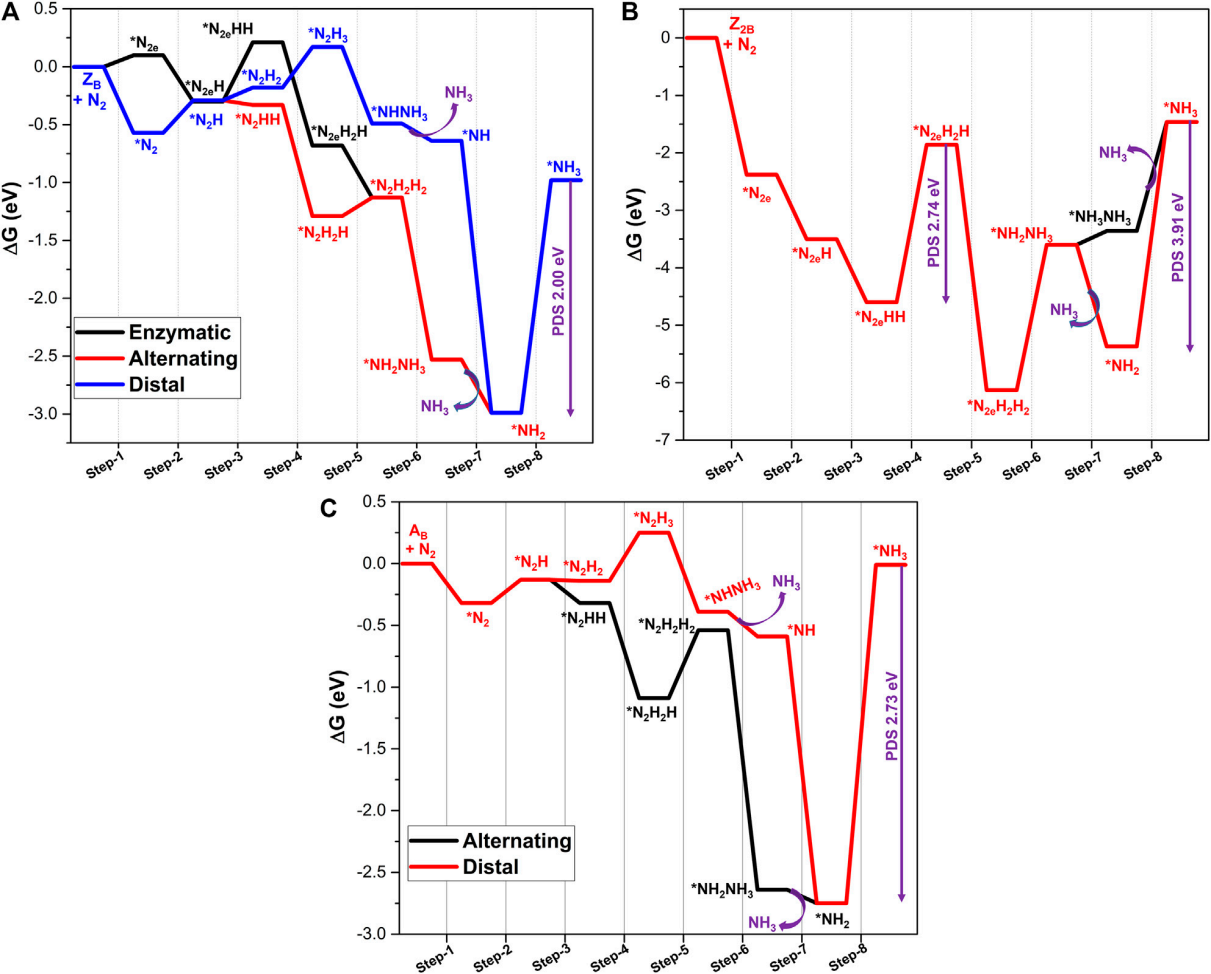
Calculated Gibbs free energy profiles of NRR catalyzed by **(A) Z**_**B**_, **(B) Z**_**2B**_, and **(C) A**_**B**_ defective systems at the PBE/6-31G(d) level of theory. PDS represents the potential determining step of the pathway.

Reduction of activated N_2_ at the B_OE_ site involves six successive protonation steps to produce ammonia. In the case of an end-on mode adsorbed system, reduction begins at the distal nitrogen site (which is away from the BNS surface) to produce the first NH_3_ molecule. Subsequently, a further attack occurs at the proximal N atom linked to the B atom, leading to the release of the second NH_3_ molecule. This reaction pathway is referred to as the “distal mechanism.” The reduction of the end-on mode of N_2_ may occur in an alternating pathway. In this pathway, both the activated nitrogen atoms are protonated in an alternative fashion to yield ammonia. Typically, the adsorption of the side-on mode occurs with the aid of an alternating pathway. The complete nitrogen reduction reaction pathways have been given in Figure 4. Adopting these reaction pathways, Gibbs free energies of all the elementary steps have been systematically calculated to unravel the minimum reaction pathway or most feasible pathway for the nitrogen reduction reaction. The results are depicted in Figure 5. The free energy profile of NRR catalyzed by the B-open edge (**Z**_**B**_) site is illustrated in Figure 5A. Since adsorption of N_2_ with **Z**_**B**_ occurs in all possible modes, distal, alternating, and enzymatic pathways have been investigated. Except for the final ammonia desorption step, the elementary step with maximum endergonicity is called the potential determination step (PDS), and the corresponding Gibbs free energy is denoted as ΔG_PDS_. In the literature, this potential is also called the limiting potential of the NRR, which describes the possibility of the nitrogen reduction reaction.

For the **Z**_**B**_ mode of absorption, the first step of hydrogenation of *N_2_ to form *N-NH is endothermic. The energy requirements for end-on and side-on modes are 0.27 and 0.11 eV, respectively. The lower endergonicity in the case of the side-on mode indicates that the possibility of activation of N_2_ is high in the side-on mode when compared to the end-on mode. Following the reduction of end-on mode-activated species, further hydrogenation provides the selectivity between distal and alternating pathways. Second protonation (step 3) leads to the formation of *N_2_H_2_ (distal) and *N_2_HH (alternating) intermediates with energetics of 0.12 and −0.03 eV, respectively. This step favors the alternating pathway over the distal pathway. Hence, an alternating pathway is further considered. In step 4, the third hydrogen is added to the distal nitrogen to form *N_2_H_2_H, and this step is downhill by −0.96 eV. Adopting the same pathway, the fourth hydrogen is added to the proximal nitrogen in step 5 to get *N_2_H_2_H_2_. In this step, the complete scission of the N-N bond is observed to form two NH_2_ adducts. This step is an endergonic one with the energetics of 0.16 eV. The subsequent fifth protonation (step 6) results in the formation of the first NH_3_ molecule at the active site and NH_2_ adduct. This step is associated with the release of high energy of −1.39 eV. It can be seen from the free energy profile that step 7, corresponding to desorption of NH_3,_ is also an exergonic reaction (−0.46 eV). Final protonation (step 8) involves the formation of a second NH_3_ molecule. However, this step is highly endergonic and turns out to be a potential determining step with energetics of 2.00 eV. In the case of the side-on mode of adsorption, the first hydrogen addition is exergonic by −0.39 eV. This exergonicity is attributed to the significant activation of N_2_ by **Z**_**B**_ in the side-on mode of adsorption. In this mode, reduction follows an enzymatic pathway where protonation occurs in an alternating fashion. The second proton addition is an uphill step with 0.50 eV followed by formation of two downhill intermediates *N_2e_H_2_H (−0.88 eV) and *N_2e_H_2_H_2_ (−0.45 eV). Interestingly, the *N_2e_H_2_H_2_ intermediate resembles the intermediate of step 5 in the alternating pathway. Hence, further reduction steps follow the alternating mechanism. Nitrogen reduction at the zigzag B-open edge demands energy due to the appreciable interaction of active site with nitrogen and stabilization of the products.

Results from calculations on nitrogen reduction at two B-open edge sites (**Z**_**2B**_) (Figure 5B) reveal that the first protonation step is downhill by −1.11 eV, due to the considerable activation of N_2_ by **Z**_**2B**_. In the subsequent reduction step (step 3), formation of *N_2_HH takes place, and the corresponding energy is −1.10 eV. In step 4, the third proton is added to the distal nitrogen to form the *N_2_H_2_H intermediate. Formation of this intermediate costs high energy of 2.74 eV. The fourth protonation gives a hydrazine adduct at the active site along with complete scission of the N_2_ bond. The completion of this step yields two NH_2_ species to the active sites. It can be seen from the energy profile that this step is associated with the favorable free energy of −4.27 eV. The fifth protonation leads to the formation of the first ammonia molecule; however, this step requires free energy of 2.53 eV. Since the interaction between the catalysts and the intermediate is considerably high in the previous step, a subsequent reduction is associated with a very high potential requirement (2.53 eV). This step is followed by the desorption of first ammonia molecule and it is related to energy of 0.24 eV. Prior to the desorption of the first ammonia molecule, the final protonation at the NH_2_ adduct gives the second ammonia molecule. In this intermediate, both the ammonia molecules are bound to the active site. It can be seen from the energy profile that it is a downhill step (−1.76 eV). The energetics indicate the favorable formation of ammonia molecules.

The complete energy profile for the various intermediates and products in the reaction as catalyzed by the armchair open B-edge site (**A**_**B**_) model is given in Figure 5C. It is clear from the figure that the first protonation is uphill by 0.19 eV, followed by the second protonation. The second protonation adopts more a feasible alternating pathway when compared to the distal pathway. The free energies associated with step 3 are −0.01 and −0.19 eV for distal and alternating pathways, respectively. Hence, further reduction occurs via an alternating pathway. The third protonation leads to the formation of *N_2_H_2_H, which is exergonic in energy by −0.77 eV. The formation of *N_2_H_2_H_2_ (step 5) is an uphill process (0.55 eV). In this step, the N-N bond is completely cleaved. After the formation of ammonia in the fifth protonation step, the energy of −2.09 eV is released. Desorption of the first ammonia molecule is a downhill process (−0.11 eV), and subsequently, protonation of the intermediate leads to the formation of the second NH_3_. It can be observed that this step is highly endergonic in nature and emerges as PDS with 2.74 eV.

### Carbon Doping at the Edges of BN

It is clear from the energetics of NRR by B-open edge systems that the active B-sites strongly interact with molecular nitrogen and intermediates of NRR. Thus, the desorption of the product from the surface is energetically demanding, and connectedly poisoning catalysts is highly possible. Hence, the activity of these open B-edges should be tuned to facilitate the NRR in an efficient manner. In this context, the catalytic performance of BN can be improved by doping.

Specifically, we have considered doping of carbon (C), sulfur (S), and phosphorous (P) on the various chosen models. However, our computational results showed that doping of S and P are energetically unsuitable for N_2_ reduction. Hence, these systems are not considered for further exploration. Analysis of literature information shows that there are abundant experimental details on the effect of carbon doping on the BN systems (Chen et al., 2018; Marbaniang et al., 2018; Almahmoud and Talla, 2019). It is found from previous studies that carbon doping can alter the catalytic properties of BN systems. Therefore, doping of carbon has been investigated in detail.

**FIGURE 6:**
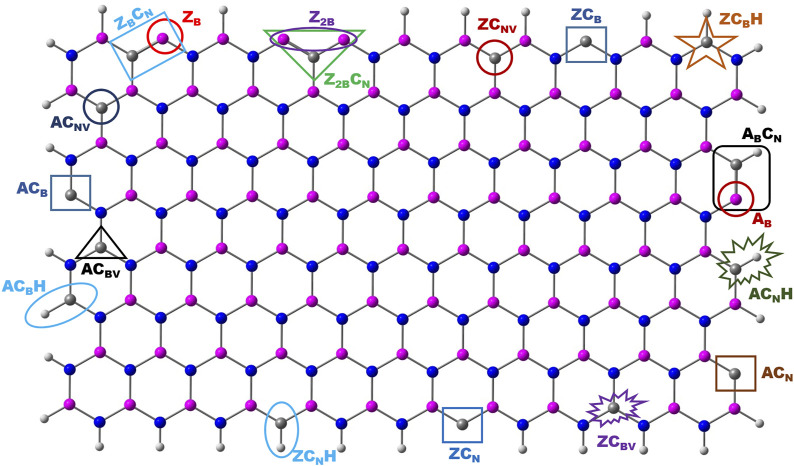
Schematic representation of various possible edge sites for carbon doping in the BN system.

**FIGURE 7:**
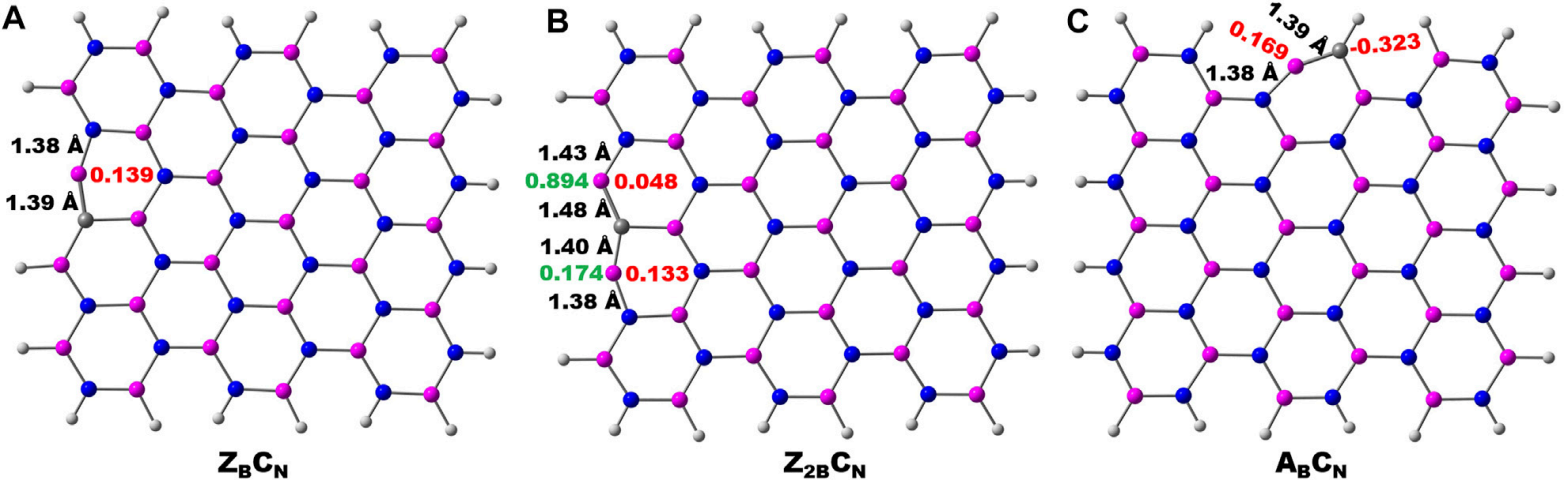
Optimized geometries of carbon doping at the N atom vicinal to the B-open edge active site calculated at the PBE/6-31G(d) level of theory. Important bond lengths around active sites are given in black-colored font. The charge and spin densities on the active site are shown in red and green-colored font, respectively.

The schematic of different possibilities of carbon doping in BN materials is shown in Figure 6. It can be noticed from the optimized geometries of carbon-doped BN systems (Figure 7) that carbon doping is carried out at the nitrogen atom, which is vicinal to the B-open edge site of **Z**_**B**_, **Z**_**2B**,_ and **A**_**B**_. These doped models are referred to as **Z**_**B**_ **C**_**N**_, **Z**_**2B**_ **C**_**N**,_ and **A**_**B**_ **C**_**N**_. The charge population analysis of these systems revealed that this strategy may regulate the charge redistribution around the B open site so that the high limiting potentials of NRR obtained for open B-active sites may be tuned significantly. Similar arguments have also been advanced in previous reports (Jiang et al., 2007; Ni and Wang, 2015; Mao et al., 2019).

There is a compelling evidence that these doped C-sites may act as active sites for possible catalytic applications. Therefore doping of the C atom at various possible positions of both zigzag and armchair edges of pristine BN may lead to new defective systems. The optimized geometries at the PBE/6-31G(d) level of theory are presented in the Supplementary Material (SM) as Supplementary Figures S1, S2. It is clear from the geometries of C-doped systems (Figure 7, Supplementary Figures S1, S2) that the edge B atoms of C-doped BN on the B-edge move in an inward direction and the associated symmetry of the BN system is lost. The optimized geometries of N_2_ adsorption modes on C-doped BN are presented in the SM (Supplementary Figures S3–S5). In this section, results from calculations on the catalytic activities of both B_OE_ and C-doped active sites at edges are presented.

### NRR at B-Open Edge Site in the Presence of C-Doping

The calculated defect formation energies of all the model systems (Figure 6) are listed in Table 1. It is clear from the energy values that these defective systems are thermodynamically possible, and it may require minimal external energy input for their creation. It is evident from the previous report that the adjustment of carbon doping within h-BN systems can be made in a more controllable fashion when compared to the incorporation of B and N in graphene (Ci et al., 2010; Huang et al., 2015). These reports have encouraged us to explore further calculations on the nitrogen reduction reaction.

**FIGURE 8:**
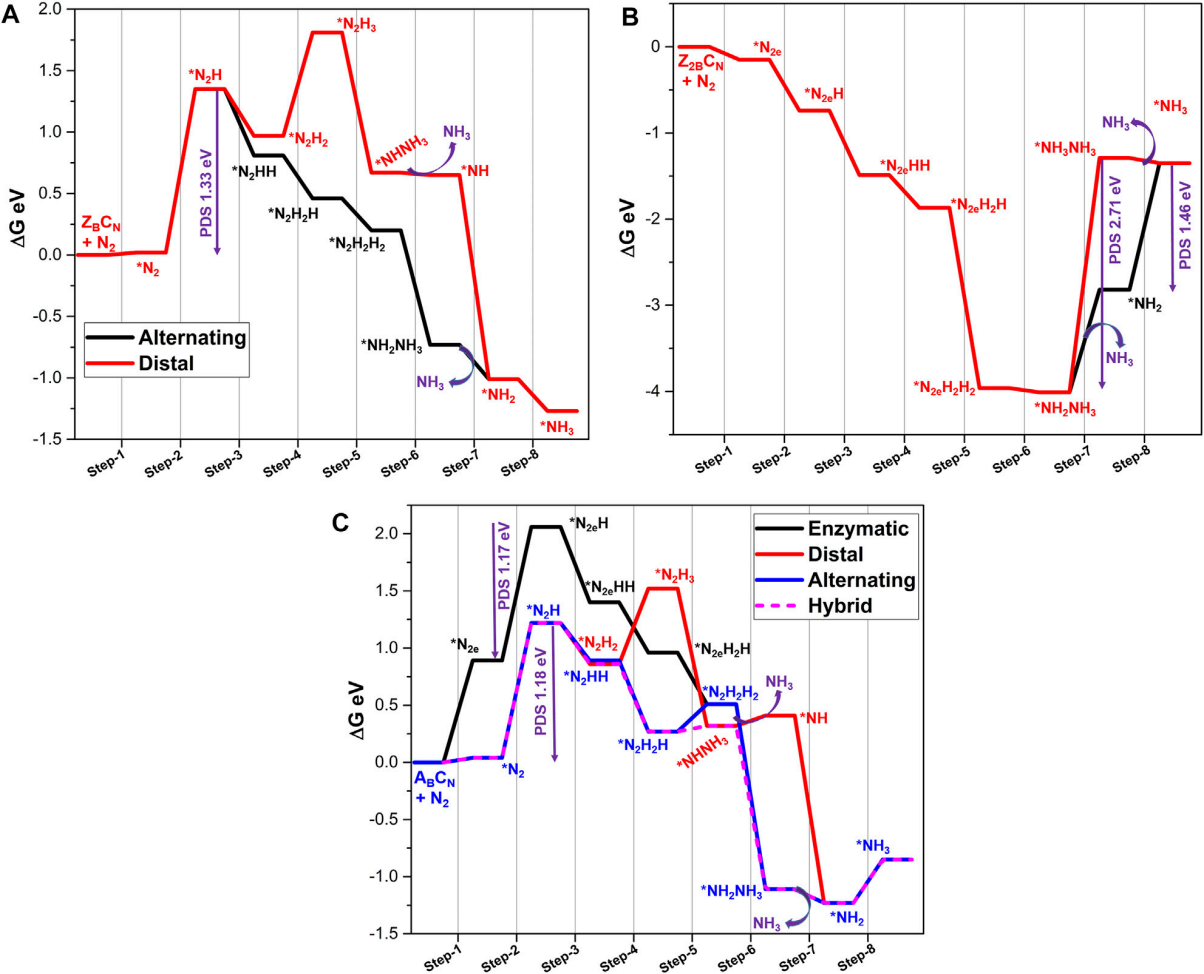
Gibbs free energy profile diagrams of possible NRR pathways catalyzed by **(A) Z**_**B**_ **C**_**N**_, **(B) Z**_**2B**_ **C**_**N**_, and **(C) A**_**B**_ **C**_**N**_ calculated at the PBE/6-31G(d) level of theory. PDS is a potential determining step and * indicates the adsorption of intermediate on to the BNS.

The free energy profiles of possible NRR catalyzed by **Z**_**B**_ **C**_**N**_, **Z**_**2B**_ **C**_**N**_, and **A**_**B**_ **C**_**N**_ are presented in Figure 8. The absorption of molecular nitrogen with **Z**_**B**_ **C**_**N**_ is studied by adopting an end-on mode of adsorption. The calculated absorption energy is (0.02 eV). Previous reports have shown that the nitrogen reduction from the marginally endergonic adsorption of N_2_ on the catalyst system is still possible (Liu et al., 2018; Tian et al., 2018). It is evident from Figure 8A that the first protonation (step 2) is again an endergonic reaction with the energetics of 1.33 eV. As explained earlier, second protonation (step 3) has possibilities for either distal or alternating pathways. It is clear from the energetics of step 3 that the alternating pathway (−0.54 eV) is more feasible. Hence, an alternating pathway is further investigated from this point. The protonation of alternating intermediate *N_2_HH yields *N_2_H_2_H in step 4. The value of free energy (−0.34 eV) shows the feasibility of this step. The formation of hydrazine can be noted in step 5. It involves the release of energy of −0.27 eV. At this particular step, possibility of a hybrid mechanism has also been studied to get *N_2_H_2_H from *NHNH_3_. However, it is endergonic in nature by 0.21 eV. Hence, an alternating mechanism is continued in the remaining step.

The formation of ammonia is seen in step 6 with the release of energy (−0.93 eV). The subsequent desorption of ammonia is favorable (−0.28 eV). The final protonation step yields a second ammonia molecule (−0.25 eV). The comparison of **Z**_**B**_ **C**_**N**_ and **Z**_**B**_ results shows that in stark contrast to NRR by **Z**_**B**_, the minimum energy pathway for **Z**_**B**_ **C**_**N**_ is favorable except for the first protonation step. This may be attributed to the presence of C-doping and associated changes in the charge distribution, which regulates the high-energy demanding steps into favorable reactions. Overall, the limiting potential decreases from 2.0 eV (in the case of **Z**_**B**_) to 1.33 eV.

The calculated energetics associated with the side-on mode of adsorption of N_2_ with C-doped defective **Z**_**2B**_ follows the same trend as observed in undoped **Z**_**2B**_. The adsorption energy is found to be -0.16 eV, which is less favorable when compared to N_2_ adsorption on **Z**_**2B**_. As a result of this adsorption, the N_2_ bond is elongated to 1.19 Å with an electron transfer of 0.26 e to the N_2_ molecule. These values are marginally less when compared to the undoped **Z**_**2B**_ system. The energy profile for this system is depicted in Figure 8B for an enzymatic pathway.

The first protonation step is associated with an energy of −0.59 eV to form the *N_2e_H intermediate. In accordance with the energetics of formation of *N_2e_HH, the next step is also favorable. The free energies of further protonation steps to form *N_**2e**_ **H**_**2**_ **H** (step 4), **N**_**2e**_ **H**_**2**_ **H**_**2**_ (step 5), and *NH_**3**_ **NH**_**2**_ (step 6) are −0.38, −2.08, and −0.05 eV, respectively. It is worth noting that both step 4 and step 6 are highly endergonic reactions in the case of **Z**_**2B**_, which are modulated to be favorable reactions in the presence of a C-doped defective site. The final protonation step to form *NH_3_NH_3_ intermediate prior to desorption of the first ammonia molecule is akin to that in **Z**_**2B**_ with the free energy of 2.71 eV. It can be seen from the free energy profile (Figure 8B) that desorption of the first ammonia molecule in step 7 is an uphill process (1.18 eV). The final reduction of *NH_2_ to form the second ammonia molecule is also an endergonic process (0.46 eV). It is clear from the results that C-doping could modulate energetics of second ammonia molecule formation, and limiting potential is reduced from 2.74 (**Z**_**2B**_) to 1.46 eV.

In the case of the C-doped armchair B-edge system (**A**_**B**_ **C**_**N**_), both end-on and side-on modes of N_2_ adsorption are possible, unlike the **A**_**B**_ system (where the only the end-on mode is observed). The adsorption energies are a non-spontaneous process, as evident from energy values. The lengthening of the N-N bond is observed in both modes along with charge transfer. Thus, in this model system, all the three reduction pathways are studied for NRR (Figure 8C). In the case of an end-on product, step 2 needs 1.18 eV for the first protonation due to the lower accumulation of charge density on N_2_ species. The second protonation is a downhill process in both distal and alternating pathways. As evident from Figure 8C, the marginal energy difference between free energies of these processes leads to problems in selectivity. Later, the alternating pathway emerged as a more feasible mechanism at the third protonation (step-4, ∆G = −0.62 eV) compared to the distal pathway (0.65 eV). The fourth protonating step again changes its pathway from alternating to the distal pathway (0.05 eV) to form the *NHNH_**3**_ intermediate. It can be seen from the energy profile that the alternating pathway results in the formation of hydrazine intermediate with an energy input of 0.24 eV. Desorption of the first ammonia molecule is endergonic in nature with the energetics of 0.09 eV. However, prior to the desorption step, the fifth protonation to *NHNH_**3**_ is a downhill process with the energetics of −1.44 eV. This step facilitates the favorable desorption of the ammonia molecule from the catalyst surface with the energetics of −0.12 eV. The final reduction step needs an energy input of 0.38 eV to form a second ammonia molecule.

The reduction of the side-on mode N_2_ in the enzymatic pathway proceeds through first protonation (1.17 eV), which is an endergonic step. Further reduction steps lead to the formation of the hydrazine adduct at step 5, which is similar to step 5 of an alternating pathway. Hence, further reductions from this intermediate exactly follow the metrics of an alternating pathway. Overall, the limiting potential of NRR is 1.17 eV, which is observed for the first protonation step in the enzymatic pathway. It is clear from the results that, without carbon doping, NRR catalyzed by **A**_**B**_ occurs with a limiting potential of 2.73 eV, whereas the same with carbon doping is reduced to 1.17 eV.

### Nitrogen Reduction at C-Doped Active Sites

In this section, the role of C-doping in the model systems as an active site for catalyzing NRR has been elucidated. The calculated geometries of these models based on carbon at different zigzag and armchair edge sites are shown in Supplementary Figures S1, S2. Both zigzag and armchair ribbons have six possible sites each for C-doping. The spin polarized DOS calculations have been carried out in order to unravel the electronic properties of the newly designed defective BNS systems and the results are given in Supplementary Figures S6, S7. It is clear from the figures that in all the carbon-doped systems, new energy states have appeared at the Fermi level. These new energy levels indicate the spin asymmetry in the defective systems which may enhance the catalytic activity of these defective systems. The feasibility of the C-active site for catalyzing nitrogen reduction has been calculated using the approach as mentioned in the previous sections.

### Carbon Doping at the Zigzag Edges

Six possible doping sites are referred to as **ZC**_**B**_ (carbon doping at the B-open zigzag edge), **ZC**_**BH**_ (carbon doping at the B-site which is H-passivated), **ZC**_**BV**_ (C-doping at the B-vacancy of the N-edge), **ZC**_**N**_ (carbon doping at the N-open zigzag edge), **ZC**_**NH**_ (carbon doping at the N-site which is H-passivated), and **ZC**_**NV**_ (C-doping at the N-vacancy of the B-edge). These sites have been taken for further investigation to reduce molecular nitrogen. In the case of **ZC**_**B**_, the open C-active site is bonded to two adjacent N atoms. This carbon is coordinately unsaturated, and it can form a total of four bonds. Hence, the adsorption of the N_2_ molecule at this particular site has been investigated. Both end-on and side-on modes of adsorption have been considered. As followed in the previous section, distal, alternating, and enzymatic pathways for reduction of molecular nitrogen have been adopted to evaluate the associated energetics. The energetics information associated with **ZC**_**B**_ is presented in Supplementary Table S1, and the free energy profile is given in Supplementary Figure S8. A strong bond formation can be observed between the C-active site and N_2_ molecule. The distance between the two systems is 1.30 Å for end-on and 1.45 Å for side-on modes. The interaction between catalysts and the N_2_ molecule is further substantiated by the favorable overlap of FMOs of model catalysts and the N_2_ molecule. The charge transfer of 0.28/0.24 e (end-on/side-on) occurs from the model catalyst surface to the N_2_ molecule. This leads to the elongation of N-N from 1.11 to 1.17 (end-on) and 1.26 Å (side-on). These geometrical and electronic features indicate the activation of N_2_ over **ZC**_**B**_. From Supplementary Figure S8, it is clear that the hybrid pathway is most feasible pathway, where the first ammonia molecule is formed at step 5 with energetics of −1.55 eV. However, the following reduction steps are uphill leading to the formation of a second ammonia molecule with over reaction energy of 0.63 eV. For the formation of the second ammonia molecule (step 8), 1.33 eV is required which makes this step a PDS. The very strong interaction of *NH species with the ZC_B_ system may be reason for the uphill potentials of step 7 and step 8 (Supplementary Figure S8).

The **ZC**_**B**_ **H** defective model system is formed by passivating the C-open site of **ZC**_**B**_ with hydrogen. A very weak end-on mode of adsorption of N_2_ is observed with the energetics of 0.31 eV, and the interaction distance is 2.87 Å. However, favorable orbital overlapping and an electron transfer of 0.06 e into π* orbital of N_2_ and marginal elongating the N-N bond (1.12 Å) confirms the activation of N_2_ by **ZC**_**B**_ **H**. The calculated results for all the steps are summarized in Supplementary Table S2, and the corresponding energy profile is presented in Supplementary Figure S9. It is clear from the results that the formation of the first ammonia molecule occurs on the fourth protonation (step 5) and is an exergonic step by −0.78 eV. The formation of a second ammonia molecule is observed to be a PDS with the energetics of 1.02 eV. Overall, the NRR by **ZC**_**B**_ **H** follows a hybrid pathway with a PDS of 1.02 eV.

**FIGURE 9:**
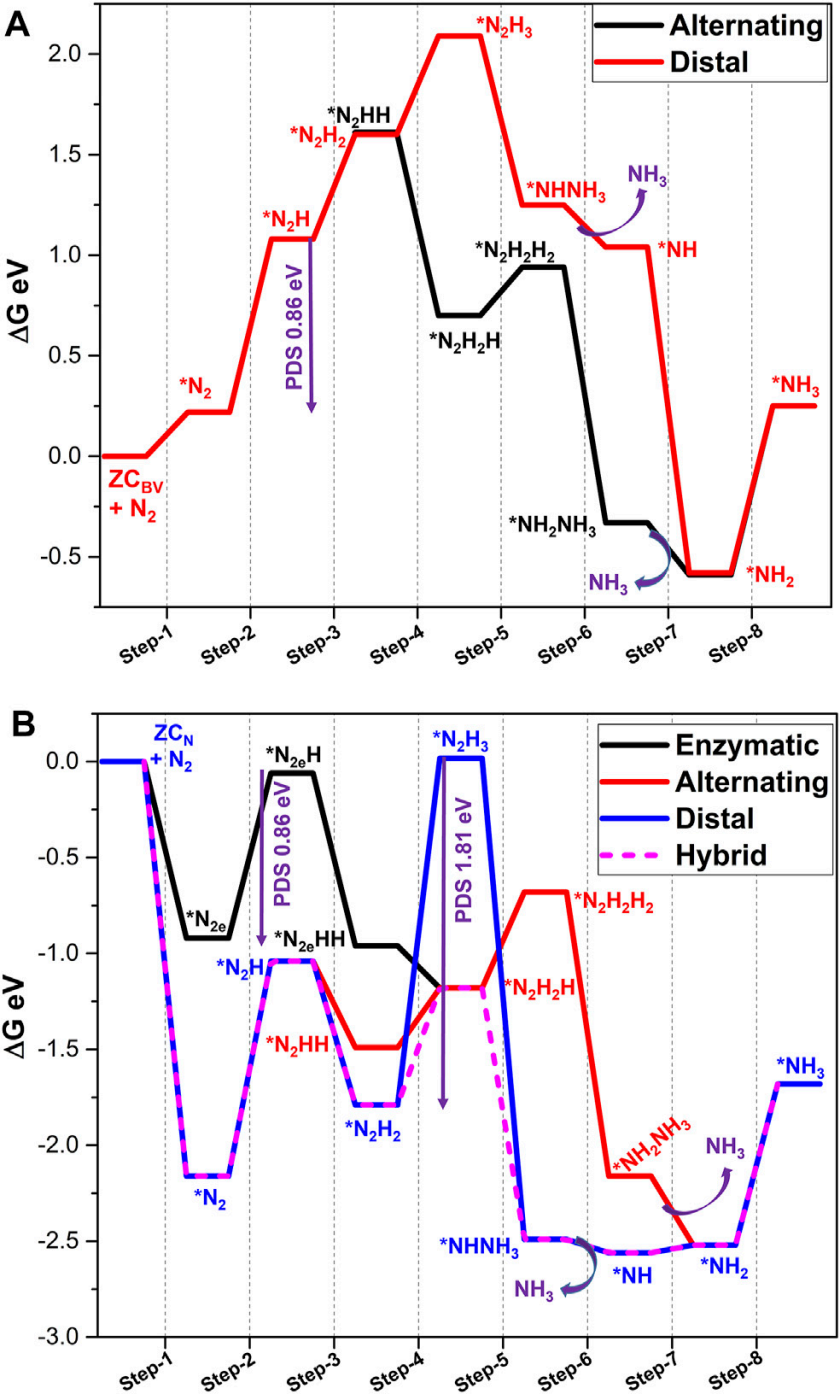
Gibbs free energy profile diagrams of possible NRR pathways catalyzed by **(A) ZC**_**BV**_, and **(B) ZC**_**N**_ calculated at the PBE/6-31G(d) level of theory. PDS is a potential determining step and * indicates the adsorption of intermediates on the catalyst.

Another defective model system, **ZC**_**BV,**_ arises due to the replacement of the B atom at the N-edge with a carbon atom. It can be observed from the geometry that the carbon atom is protruded slightly outwards from the BN lattice. Due to the high electronegativity of the three vicinal nitrogen atoms, carbon has lost 0.44 e to the model catalyst during the creation of this defective system. Further, C carries a spin density of 0.65. These geometrical and electronic parameters may induce catalytic activity to this particular site. On interaction with the N_2_ molecule, only the end-on type of adsorption is observed where the N_2_ molecule is located at a distance of 2.60 Å with interaction energy of 0.22 eV. In this process, N_2_ gains 0.17 e electrons from **ZC**_**BV**_. The mechanistic aspects of ammonia formation catalyzed by **ZC**_**BV**_ are provided in Supplementary Table S3 and Figure 9A. Like the earlier two cases, **ZC**_**BV**_ also follows a hybrid mechanism where the distal pathway dominates until step 3, and the alternating pathway becomes more feasible. Interestingly, **ZC**_**BV**_ catalyzes NRR with a lower PDS of 0.86 eV. Steps related to the formation of the first and second ammonia molecules occur with the energetics of 0.23 and 0.84 eV, respectively.

Nitrogen activation by **ZC**_**N**_ is observed in all the possible end-on and side-on modes. **ZC**_**N**_ arises by doping C atoms at the open N-edge site, which comprises two C-B bonds and is coordinately unsaturated. The C atom of this active site withdraws 0.12 e from BN and possesses a spin density of 0.23. Adsorption of N_2_ on **ZC**_**N**_ results in very strong binding with interaction energies of −2.16 (end-on) and −0.92 eV (side-on). The electron transfer from the catalyst to N_2_ molecule is observed as 0.13 (end-on) and 0.21 e (side-on). Therefore, the N-N bond is elongated to 1.15 and 1.22 Å, respectively.

Further, the details of subsequent reduction steps are given in Supplementary Table S4 and Figure 9B. Results indicate that the end-on mode of adsorption of N_2_ with **ZC**_**N**_ follows a hybrid pathway as observed in previous systems. The PDS observed for this pathway is the first protonation step with the energetics of 1.13 eV. However, reduction of the side-on mode model emerged as a more feasible pathway with PDS (step 2, 0.86 eV), which is lower than the hybrid pathway. Analysis of results reveals that the formation of the first ammonia molecule is observed as the PDS on the fifth protonation with the energetics of –2.38 eV, followed by the formation of the second ammonia molecule with energy requirements of 0.84 eV. Overall, carbon doping at the zigzag edge of BN systems **ZC**_**BV**_ and **ZC**_**N**_ can convert N_2_ to ammonia. Overall, the enzymatic pathway dominates when compared to all other possibilities.

### Carbon Doping at the Armchair Edges

Like zigzag BN, C-doping at armchair edges also resulted in six different defective BN model systems (Supplementary Figure S2). Among these, three models arise from doping of the C atom at different B-edge sites. In the other three models, doping of C at various N-edge sites has been completed. The nomenclature adopted for these systems is analogous to the zigzag defective systems. **AC**_**B**_ indicates replacement of the B atom at the armchair edge with the carbon atom to obtain a C-open edge defective site, **AC**_**B**_ **H**: arising from the doping B-edge of an armchair with C model, and is passivated with hydrogen, **AC**_**BV**_: carbon-doped at the boron site which is connected to three nitrogen atoms, **AC**_**N:**_ the carbon atom is doped at the nitrogen site of the armchair edge, **AC**_**N**_ **H**: the carbon atom is doped at the nitrogen site of armchair edge, and it is passivated by H, and **AC**_**NV**_: C-doping at the N-vacancy of the B-armchair edge.

It is clear from the results that **AC**_**B**_ and **AC**_**N**_ systems exhibit adsorption of nitrogen in both end-on and side-on modes. Hence, the subsequent reduction has been investigated by using distal, alternating, and enzymatic pathways. On the other hand, the remaining models show an only end-on mode of adsorption. In these models, only distal and alternating NRR pathways have been explored. The FMO analysis characterizes the nitrogen activation ability of these catalysts, and charge transfer into the π* orbitals of the N_2_ molecule and N-N bond elongation.

**FIGURE 10:**
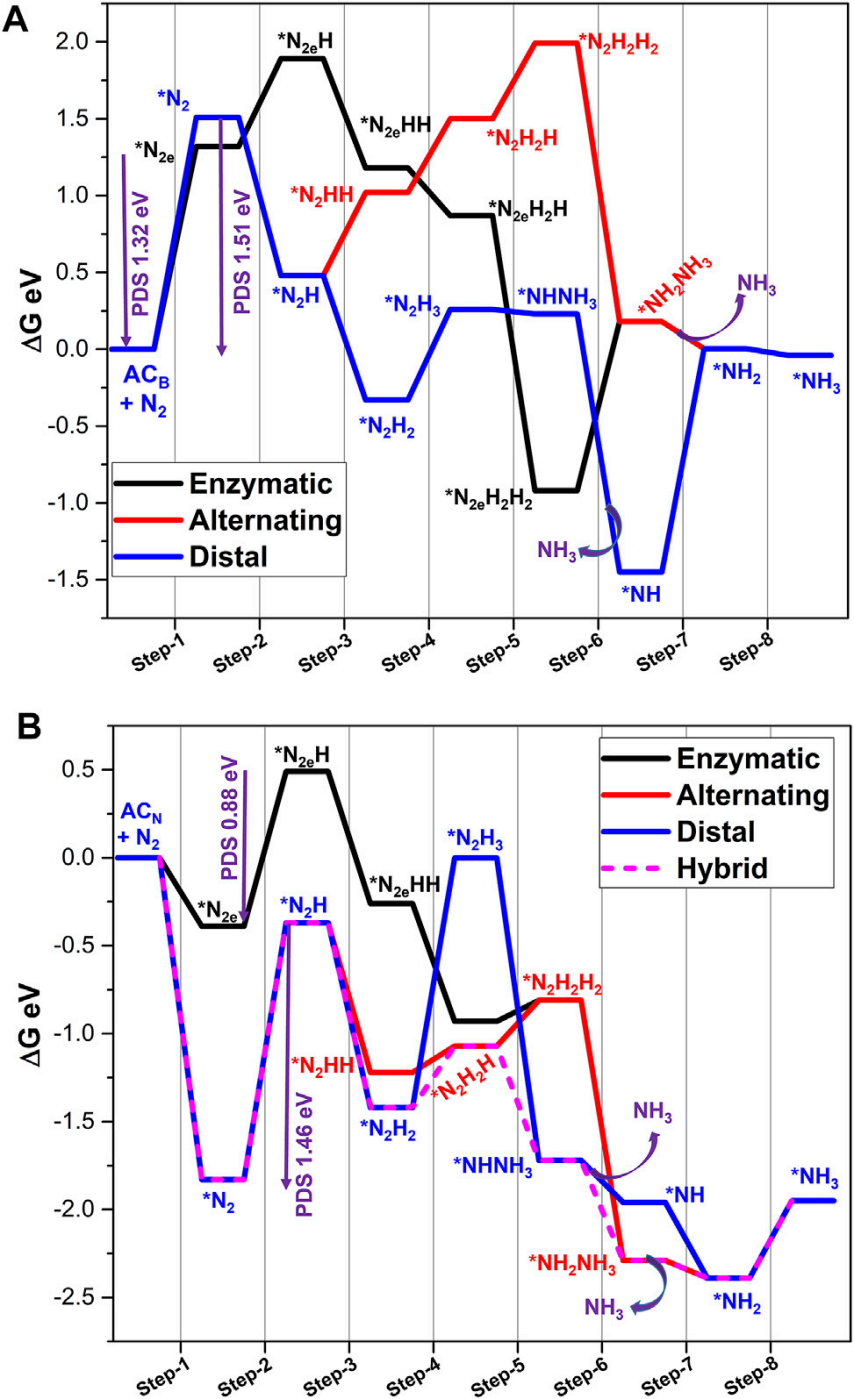
Gibbs free energy profile diagrams of possible NRR pathways catalyzed by **(A) AC**_**B**_, and **(B) AC**_**N**_ calculated at the PBE/6-31G(d) level of theory. PDS is a potential determining step and * indicates the adsorption of intermediates on the catalyst.

Further, Gibbs free energies of reaction pathways of nitrogen reduction catalyzed by these systems are listed in Supplementary Tables S5–S9, and the energy profiles are depicted in Figure 10 and Supplementary Figures S10–S12 (SM). Scrutiny of the reaction of all the energy profiles reveals that the enzymatic pathway is a dominating mechanism for **AC**_**N**_ with a lower PDS of 0.88 eV, which makes this model highly suitable for NRR when compared to all the other models of armchair edge-doped systems. In the case of adsorption of N_2_ in the end-on mode on **AC**_**N,**_ the first protonation step is observed to be PDS (0.88 eV). The formation of the first ammonia molecule is an exergonic sixth protonation step (−2.29 eV), while the formation of the second ammonia molecule demands energy of 0.44 eV. Overall, NRR catalyzed by **AC**_**N**_ is spontaneous with energy of −1.95 eV. On the other hand, the remaining model catalysts adopt a hybrid pathway to attain a minimum energy pathway. However, they exhibit higher PDS than **AC**_**N**_.

Overall, carbon doping at both zigzag and armchair edges resulted in 12 new defective model systems. Scrutiny of their ability to catalyze the NRR reveals that **ZC**_**BV**_, **ZC**_**N**_, and **AC**_**N**_ show appreciable activity among all the other catalysts. In **ZC**_**N**_ and **AC**_**N**_, the enzymatic pathway emerges as the dominating one, whereas, for **ZC**_**BV**_, NRR adopts a hybrid pathway with a lower PDS of 0.86 eV. It is also clear from the salient findings that carbon-doped model systems produced lower PDS than the B-open sites. On careful observation of all the energy profile diagrams of NRR, it is clear that NRR by some of the BNS systems is exothermic and is endothermic in the case of a few systems. For instance, the NRR catalyzed by **ZC**_**B**_, **ZC**_**B**_ **H**, **ZC**_**BV**_, **AC**_**BV**_, **AC**_**NV,**_ and **AC**_**B**_ **H** is endothermic in nature. This may be due to the non-spontaneous nitrogen activation by all these catalysts, which is a potential demanding step (endothermic). In addition, poor nitrogen activation may lead to the high potential demanding first reduction step. These two steps are contributing to the endothermic nature of NRR in these systems. Overall, **AC**_**N**_ and **ZC**_**N**_ are the two prominent catalysts, where they catalyze the nitrogen reduction reaction with lower PDS of 0.88 and 0.86 eV in highly exothermic reaction pathways of total energies −1.95 and −1.68 eV, respectively.

### Ammonia Desorption

Desorption of ammonia is one of the important parameters in nitrogen reduction. If the interaction of ammonia with the active site is very strong, then it is very difficult to desorb the ammonia molecule and it leads to either catalyst poisoning or disintegration of the ammonia molecule. To ensure such activity of the BNS systems, desorption of ammonia that is formed on the final elementary step has been investigated [∆G_des_ = (∆G_BNS_ + ∆G_NH3_)−∆G_BNS-NH3_] and the results are given in Table 2. It is clear from the table that most of the catalysts that are considered are good nitrogen reduction catalysts as they exhibit lower ammonia desorption energies (<0.86 eV, lowest PDS observed for NRR). The results indicate that the ammonia desorption step may not be the PDS for NRR catalyzed by BNS systems.

**TABLE 2:** Energetics of ammonia desorption step catalyzed by all the BNS systems.

S. No	System	∆G_des_ (eV)	S. No	System	∆G_des_ (eV)	S. No	System	∆G_des_ (eV)
1	Z_B_	0.35	6	A_B_C_N_	0.83	11	AC_B_	0.27
2	Z_2B_	1.44	7	ZC_B_	0.14	12	AC_B_H	0.09
3	A_B_	0.89	8	ZC_B_H	0.33	13	AC_BV_	0.33
4	Z_B_C_N_	1.25	9	ZC_BV_	0.27	14	AC_N_	0.69
5	Z_2B_C_N_	1.34	10	ZC_N_	0.65	15	AC_NV_	0.37

### Role of C-Doping

**FIGURE 11:**
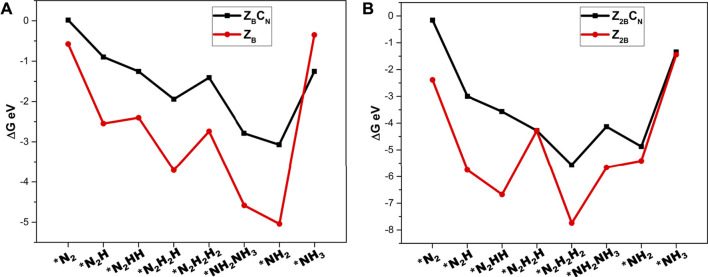
Comparison of adsorption free energies of minimum energy pathways followed for NRR catalyzed by **(A) Z**_**B**_ Vs. **Z**_**B**_ **C**_**N**_ and **(B) Z**_**2B**_ Vs. **Z**_**2B**_ **C**_**N**_ for unraveling the role of carbon doping calculated at the PBE/6-31G(d) level of theory.

In order to gain insight into the role of carbon doping in regulating the potentials of NRR, the comparison of the energetics of various intermediates in the models with and without carbon doping has been made in Figure 11.

It can be found from Figure 11 that the adsorption energies of the intermediates decrease until the formation of the first ammonia molecule and subsequently increase after the formation of the second ammonia molecule. Furthermore, the intermediates of NRR interact more strongly with B-open edge sites when compared to interaction with B-open edges with C-doping. These findings indicate that C-doping weakens the binding strength of intermediates with the surface and decreases the chance of catalyst poisoning by the strong adsorption of intermediates on the surface of the model catalyst. It is interesting to note that carbon doping has a positive role in modulating the charge density distribution of the model catalyst surface and lowering the lower potential requirements.

As stated in the previous sections, each model system favors one reaction pathway. Among them, C_N_ open sites at the nitrogen edge of BN (both **ZC**_**N**_ and **AC**_**N**_) and **C**_**B**_ active sites of **ZC**_**BV**_ exhibit highly appreciable activity towards nitrogen reduction with the lower limiting potentials of 0.86, 0.88, and 0.86 eV, respectively. The complete nitrogen reduction reactions of the earlier two models (**ZC**_**N**_ and **AC**_**N**_) are energy-efficient. However, NRR by **ZC**_**BV**_ demands external energy for the reaction. These results are comparable with the recent reports on NRR by various catalysts such as boron-doped graphene systems (0.43–1.30 V) (Yu et al., 2018), H-BN nanomaterials (0.75 V) (Zhang et al., 2019), V_3_C_2_ MXenes (0.64 eV) (Azofra et al., 2016), and defect-free MoS_2_ (0.68 eV) (Li et al., 2018).

### Selectivity Between HER and NRR

**FIGURE 12:**
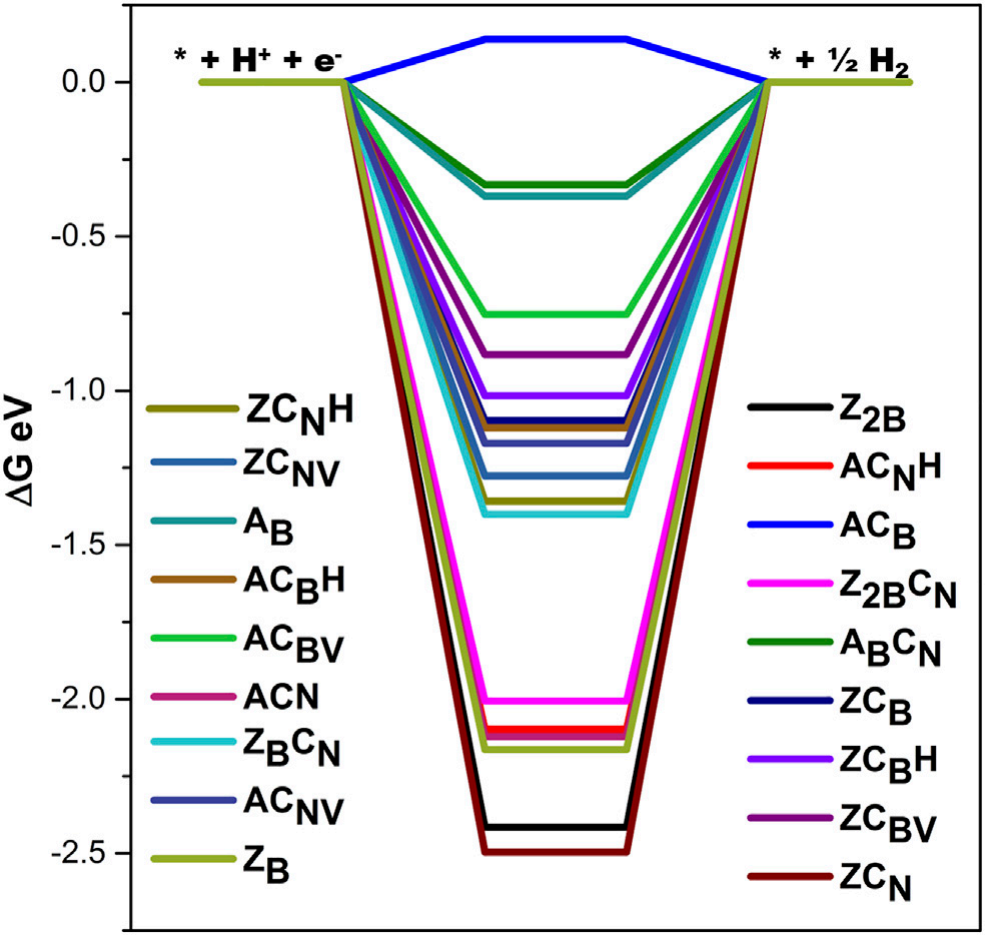
Free energy profile for hydrogen evolution reaction energetics of defective BN systems considered in this study calculated at the PBE/6-31G(d) level of theory.

It is clear from the above discussions that carbon doping significantly enhances the catalytic ability of BN edge sites for nitrogen reduction. However, the competition for the hydrogen evolution reaction (HER) needs to be explored to assess the NRR ability of the model catalyst. As HER is competing with the reaction conditions of NRR, edge defective BN systems have also been investigated for HER ability. The calculated free energy profiles for different models are depicted in Figure 12. In general, the ideal Gibbs free energy of H adsorption for HER catalysts (ΔG_*H_) is nearly 0.0 eV. It is clear from the present results that all the models exhibit favorable ∆G values for H adsorption except **AC**_**B**_. Results demonstrate that carbon-doped zigzag BN systems are more efficient in inhibiting HER when compared to armchair counterparts. It is also evident that C-doping significantly weakens the absorption of H on edge B atoms of the zigzag BN system. Hence, most of the model systems considered in this study are good candidates for NRR by inhibiting HER.

## Conclusion

In summary, a systematic investigation on the nitrogen reduction feasibility of B-open edge defective BN nanomaterials has been carried out. The effect of carbon doping on the catalytic properties of these B-sites has been assessed. The most favorable pathways adopted by the model systems studied are summarized in Table 3. The significant findings are summarized as follows:

1) Most of the models of edge sites with and without carbon doping activate nitrogen by either strong covalent interactions or weak non-covalent interactions.
2) Carbon doping significantly enhances the catalytic activity of B-open edge sites by modulating charge density distribution and reducing the limiting potential.
3) Carbon doping effectively decreases the interactions between catalysts and NRR intermediates and concomitant reduction in the poisoning of the model catalyst reducing the catalytic activity of edges towards nitrogen fixation.
4) Overall, carbon doping plays a positive role in modulating the catalytic ability of boron edge sites.

**TABLE 3:** The summary of the minimum energy pathways followed by all the model systems.

S. No	Model system	Minimum energy pathway	Remarks
1	**Z**_**B**_	Alternating pathway	PDS is the final protonation step with energetics of 2.00 eV
2	**Z**_**2B**_	Enzymatic pathway	PDS is the third protonation step to form *N_2_H species with endergonicity of 2.74 eV
3	**A**_**B**_	Alternating pathway	PDS is the final protonation step with energetics of 2.73 eV
4	**Z**_**B**_ **C**_**N**_	Alternating pathway	First protonation step as PDS with energetics of 1.33 eV
5	**Z**_**2B**_ **C**_**N**_	Enzymatic pathway	Sixth protonation as PDS with 1.45 eV
6	**A**_**B**_ **C**_**N**_	Hybrid pathway	First protonation as PDS of 1.18 eV
7	**ZC**_**B**_	Hybrid pathway	Second ammonia formation as PDS of 1.33 eV
8	**ZC**_**B**_ **H**	Hybrid pathway	Second ammonia formation as PDS of 1.02 eV
9	**ZC**_**BV**_	Alternating pathway	PDS is the first protonation with 0.86 eV. Lowest among all the investigated catalysts
10	**ZC**_**N**_	Enzymatic pathway	First protonation as PDS with 0.88 eV
11	**ZC**_**N**_ **H**	---	No feasible interaction with N_2_
12	**ZC**_**NV**_	---	No feasible interaction with N_2_
13	**AC**_**B**_	Hybrid pathway	Activation of nitrogen in enzymatic mode is PDS with 1.32 eV
14	**AC**_**B**_ **H**	Hybrid pathway	Second ammonia formation step as PDS with 0.91 eV
15	**AC**_**BV**_	Hybrid pathway	First protonation step as PDS with 1.14 eV
16	**AC**_**N**_	Enzymatic pathway	First protonation with 0.88 eV as PDS
17	**AC**_**N**_ **H**	---	No feasible interaction with N_2_
18	**AC**_**NV**_	Hybrid pathway	First protonation step as PDS with 1.32 eV

Since it is possible to create doped edge sites in h-BN experimentally, the present investigation clearly opens a new paradigm for the development of novel NRR catalysts. Thus, the edges of BN sheets may be successfully explored for the development of BN-based catalysts by suitable doping as explained in the above discussion.

## Data Availability

The original contributions presented in the study are included in the article/Supplementary Material, further inquiries can be directed to the corresponding author.
