# Supplementary material for: Nitrogen Fixation at the Edges of Boron Nitride Nanomaterials: Synergy of Doping
Source: Front Chem. 2022 Jan 21;9:799903. doi: 10.3389/fchem.2021.799903 (PMC8814371; doi:10.3389/fchem.2021.799903)
Supplement: Supplementary file 1 [file DataSheet1.docx]

**Supplementary Material**

Nitrogen Fixation at the Edges of Boron Nitride Nanomaterials: Synergy of Doping

Venkata Surya Kumar Choutipalli^1,2,3^ Karthikraja Esackraj^2,3^ and Venkatesan Subramanian*^,1,2,3^

*^1^Inorganic and Physical Chemistry Laboratory, CSIR-Central Leather Research Institute, Adyar, Chennai-600 020, India.*

*^2^Centre for High Computing, CSIR-Central Leather Research Institute, Adyar, Chennai-600 020, India.*

*^3^Academy of Scientific and Innovative Research (AcSIR), Ghaziabad-201 002, India.*

***E–mail**: [subuchem@hotmail.com](mailto:subuchem@hotmail.com), [subbu@clri.res.in](mailto:subbu@clri.res.in).


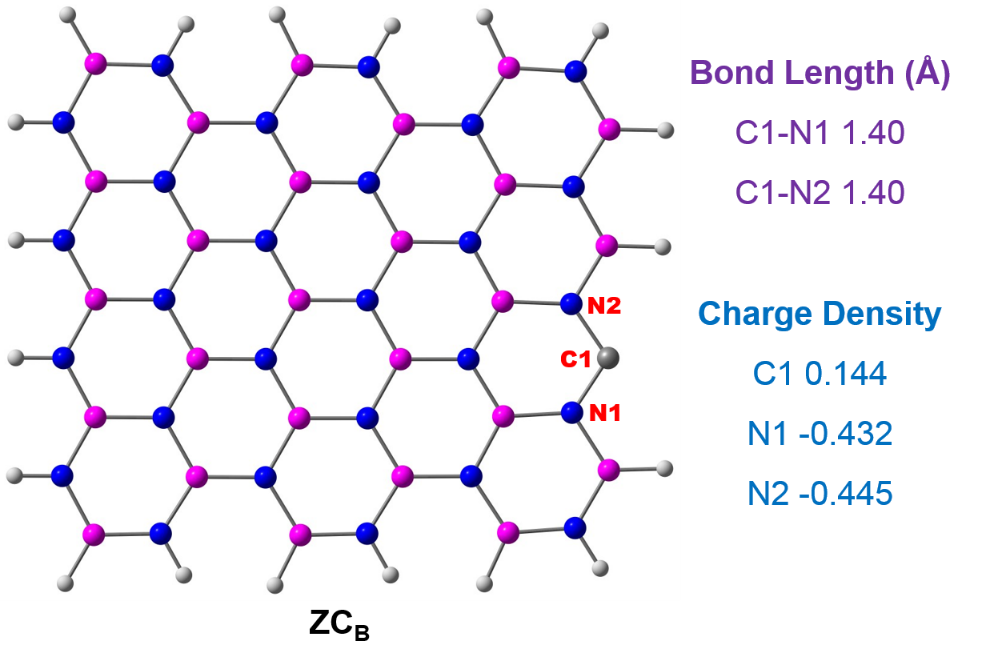


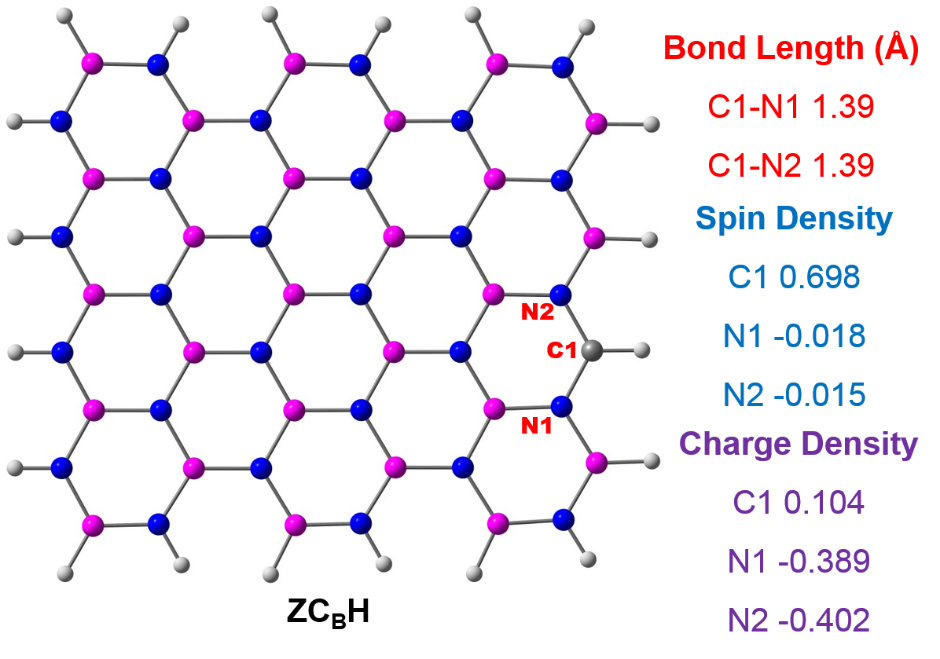


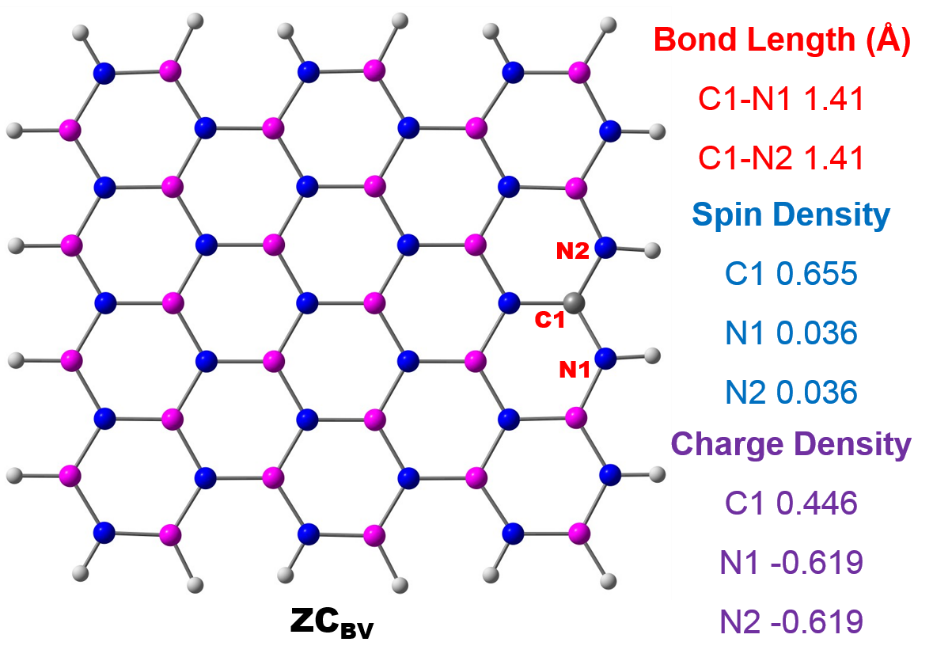


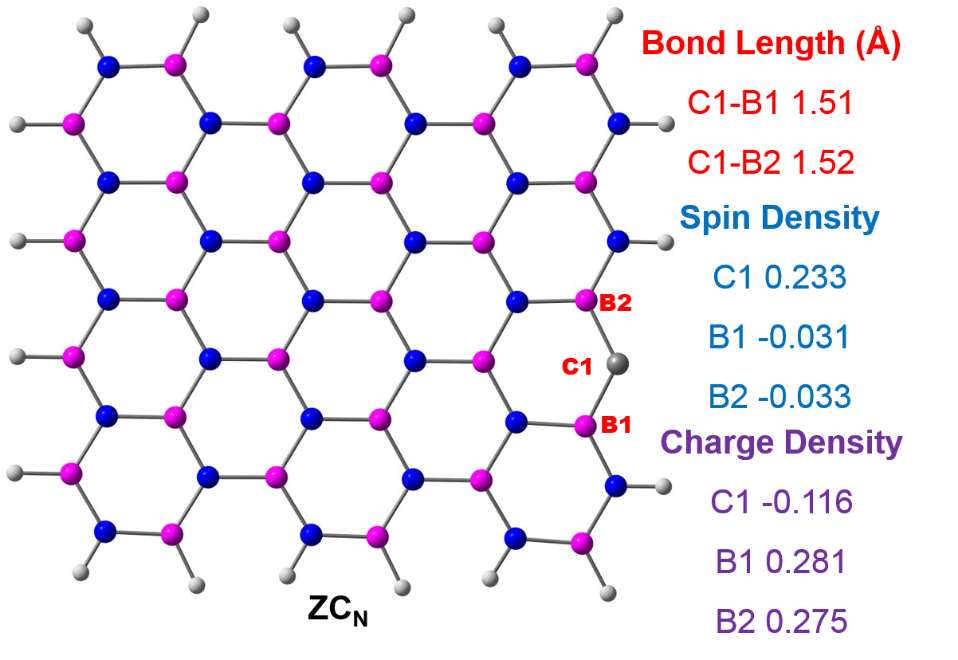


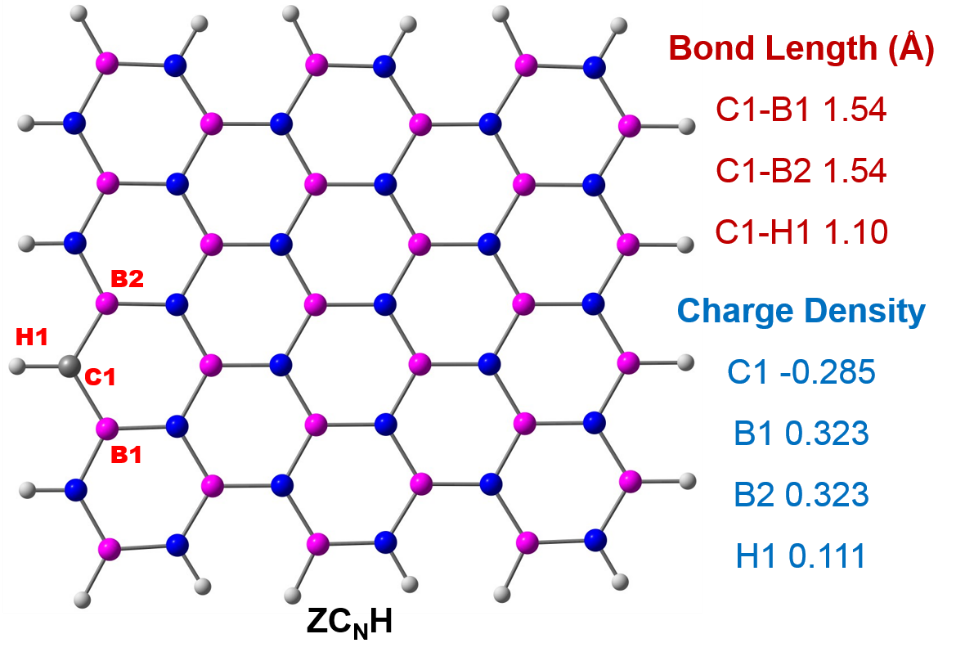


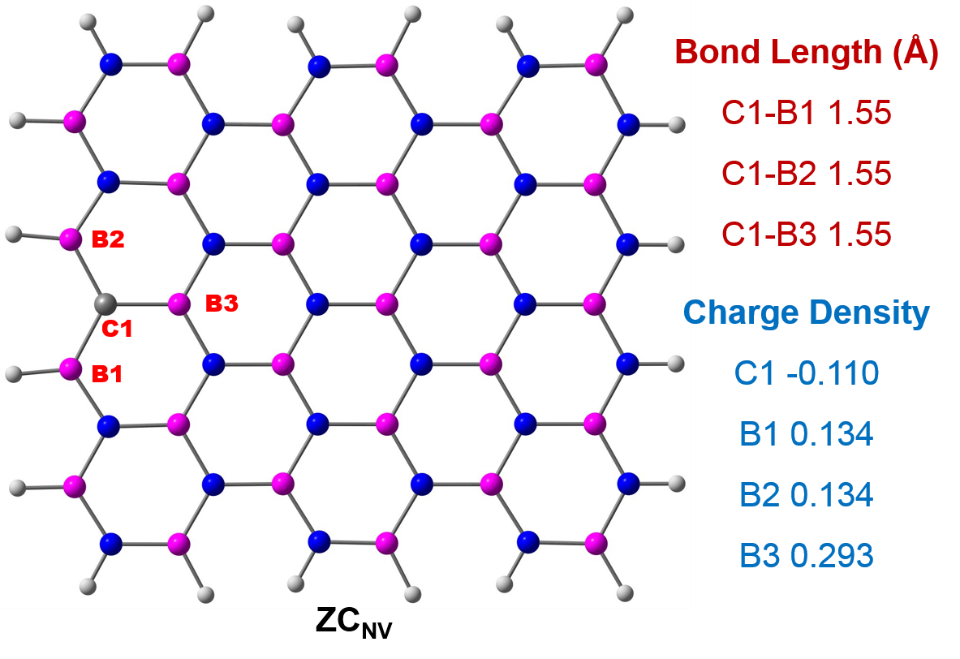


**Figure S1**. Optimized geometries of defective systems modeled by doping carbon at various zigzag edge sites calculated at PBE/6-31G(d) Level of Theory.


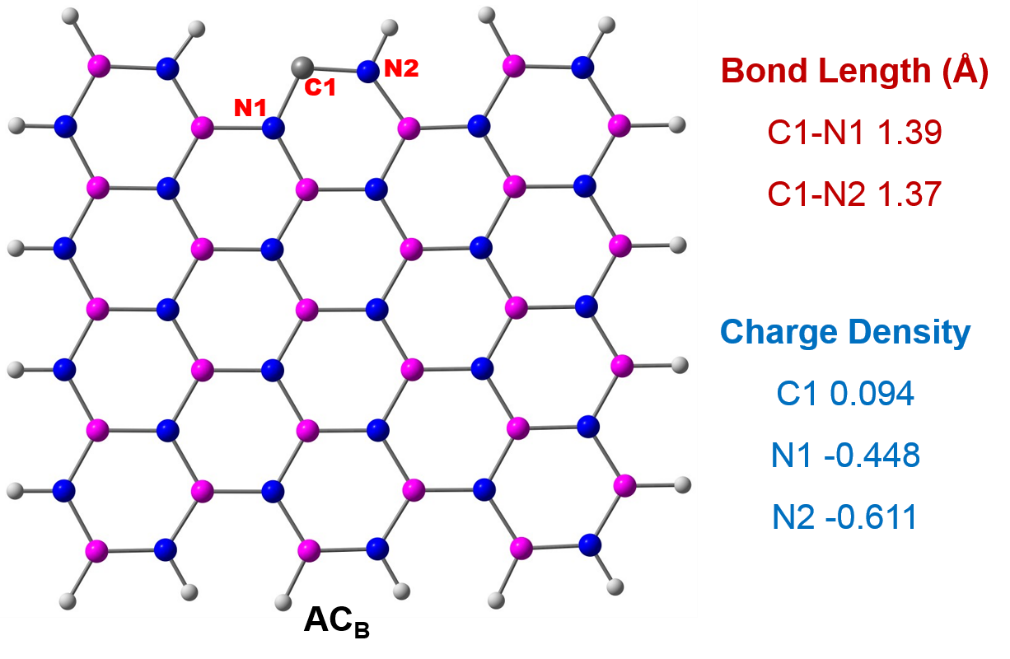


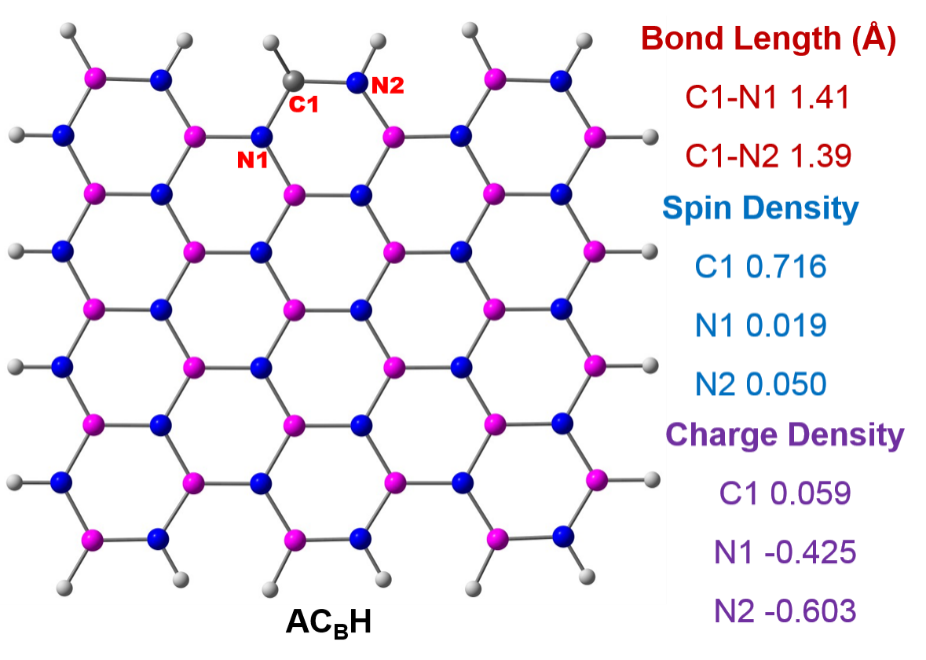


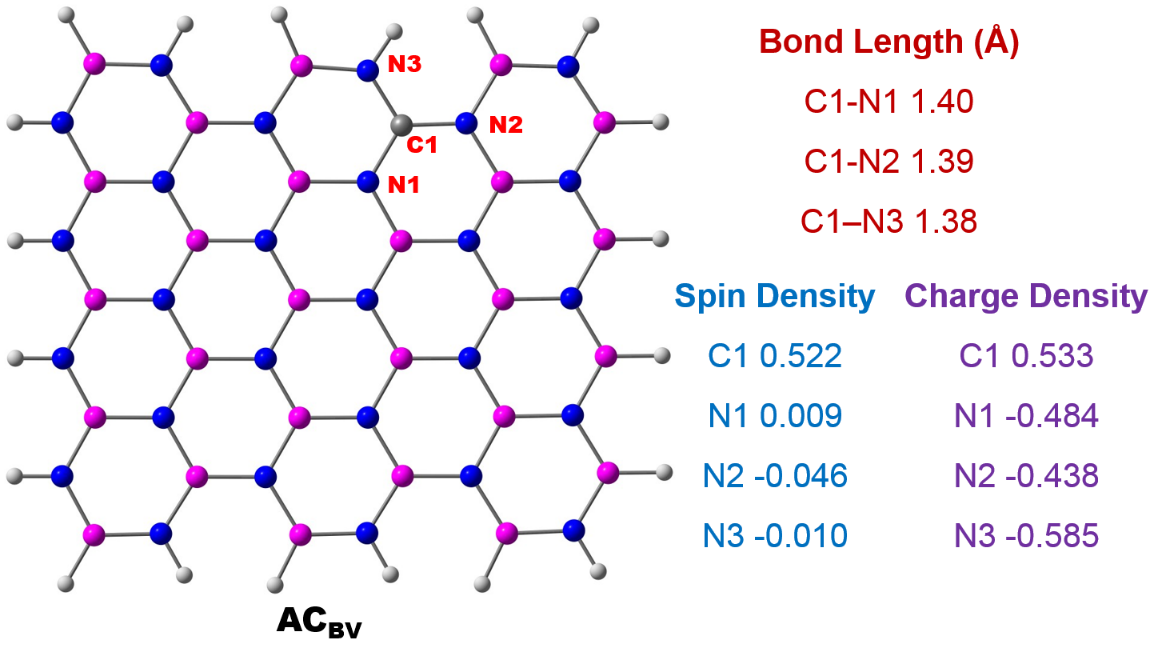


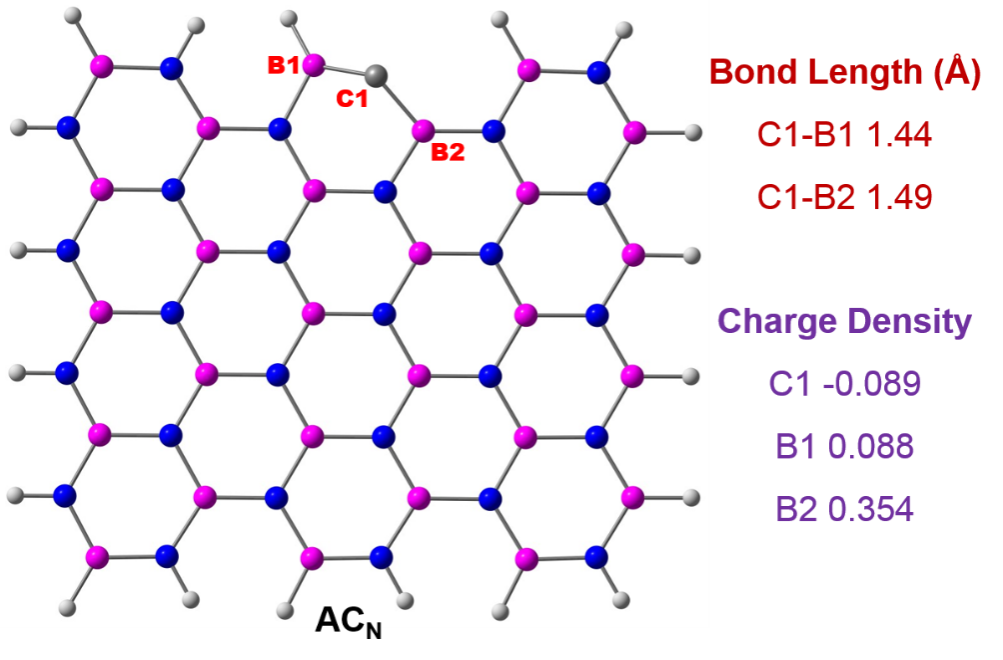


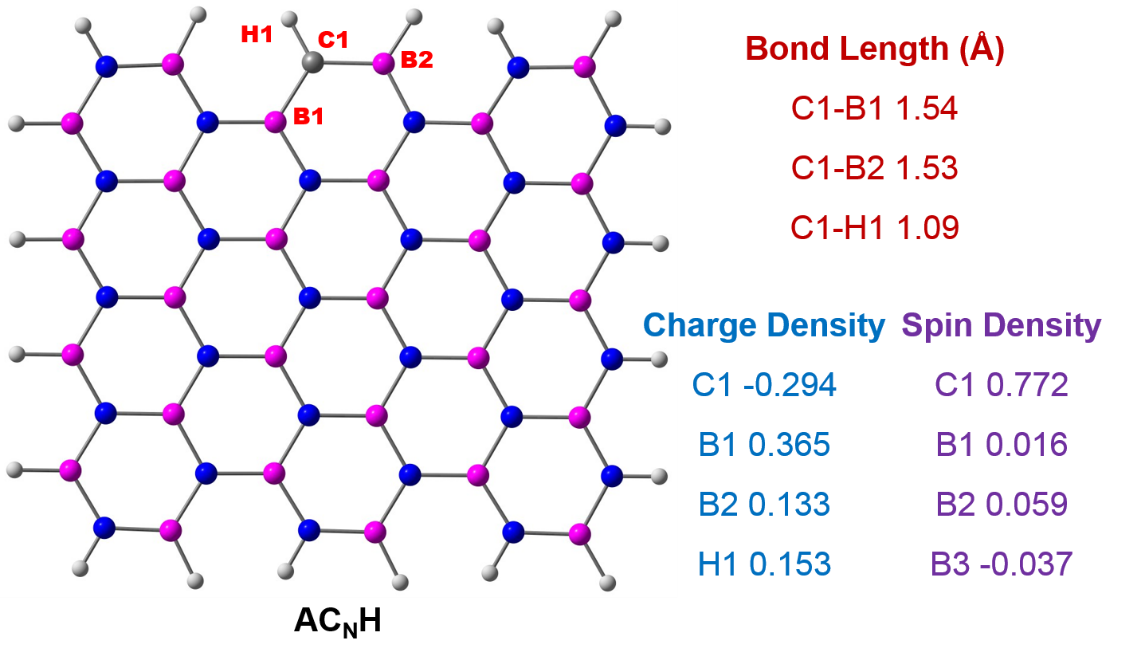


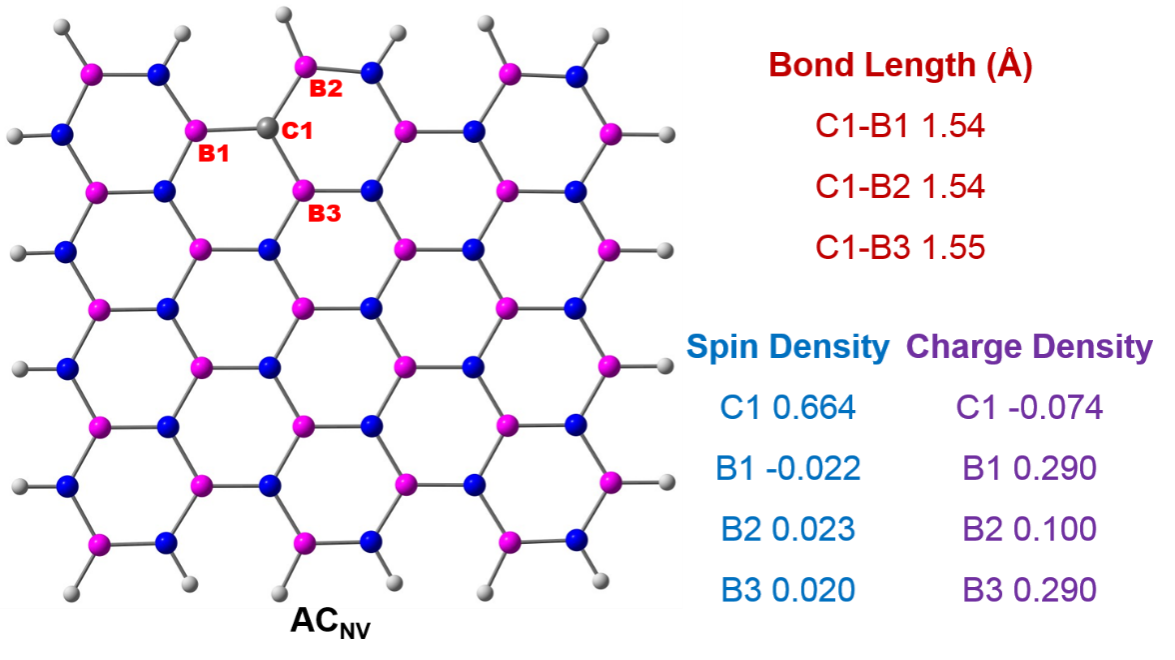


**Figure S2**. Optimized geometries of defective systems modeled by doping carbon at various armchair edge sites calculated at PBE/6-31G(d) Level of Theory.


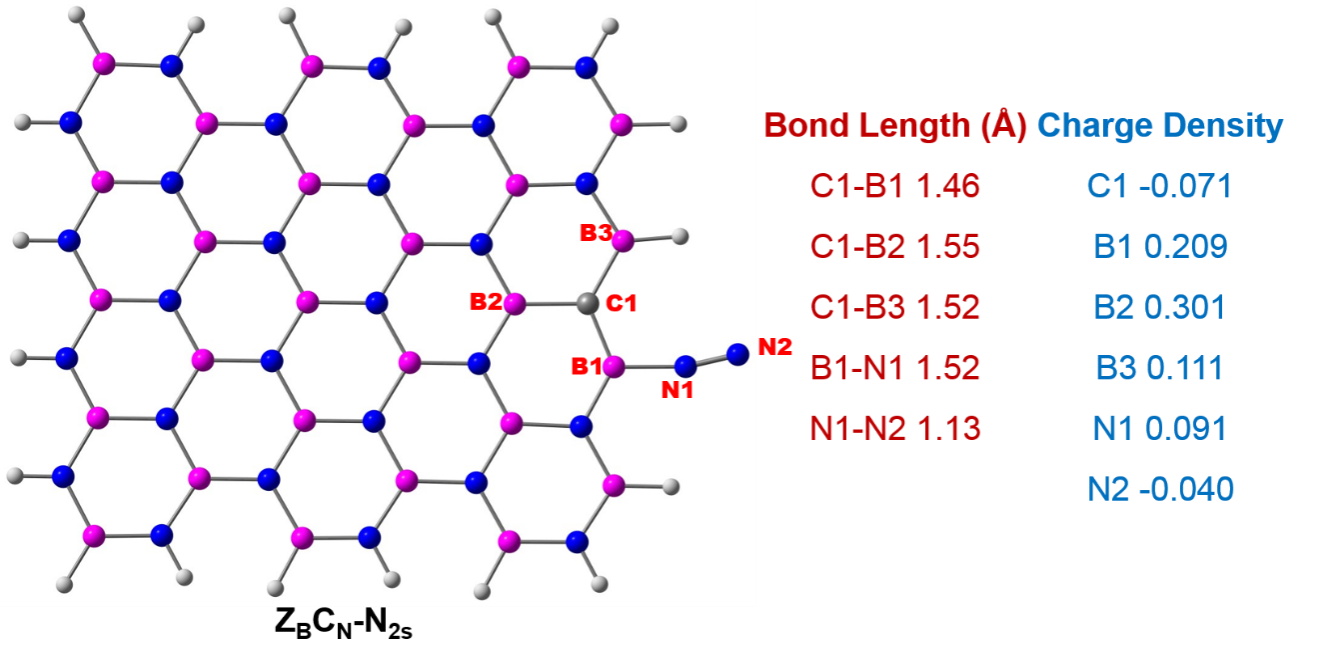


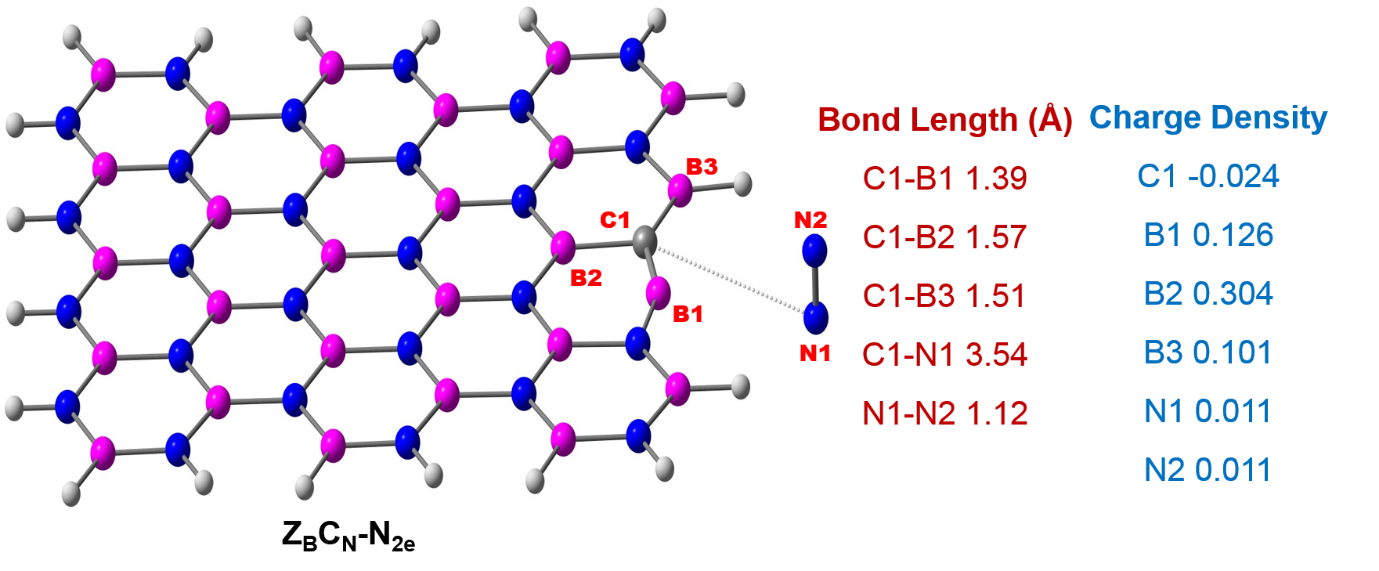


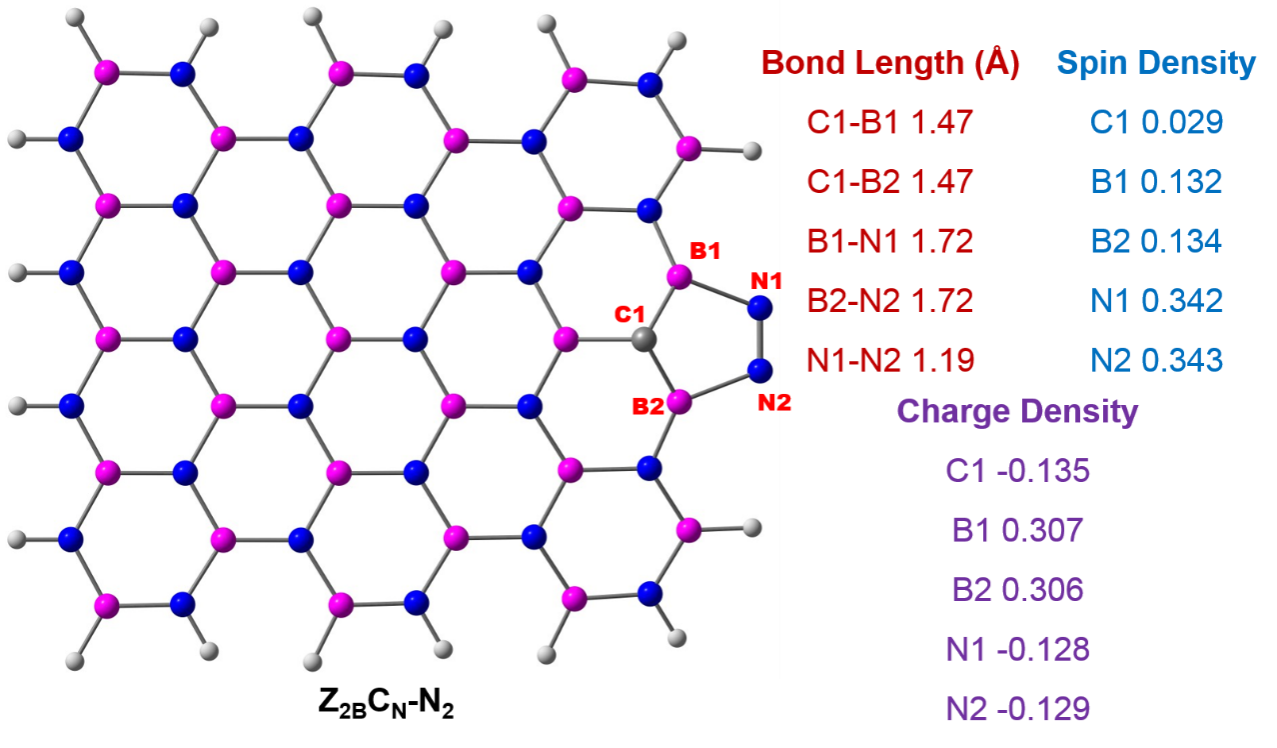


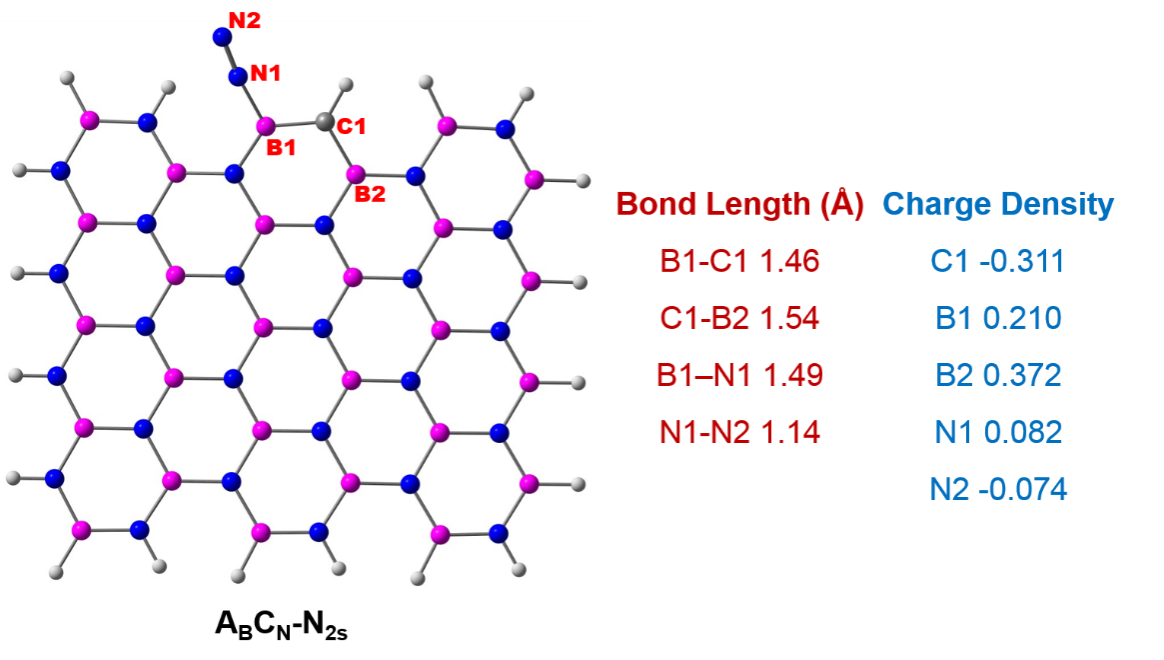


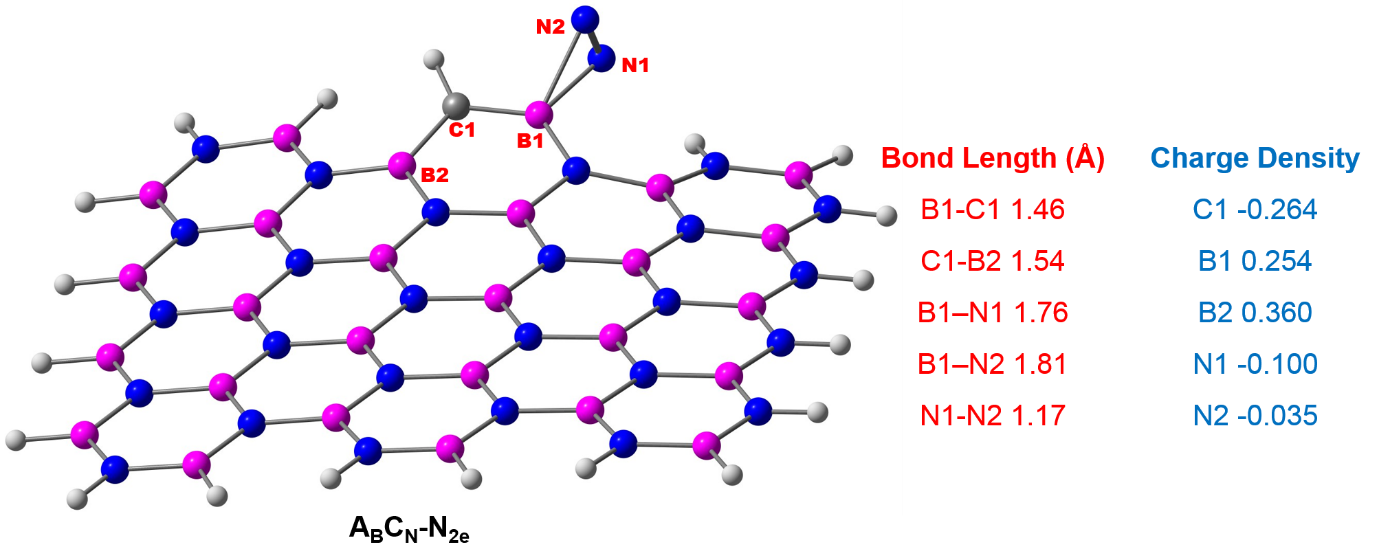


**Figure S3**. Optimized geometries of N_2_ adsorption on defective systems modeled by doping carbon at vicinal to the B-open sites calculated at PBE/6-31G(d) Level of Theory.


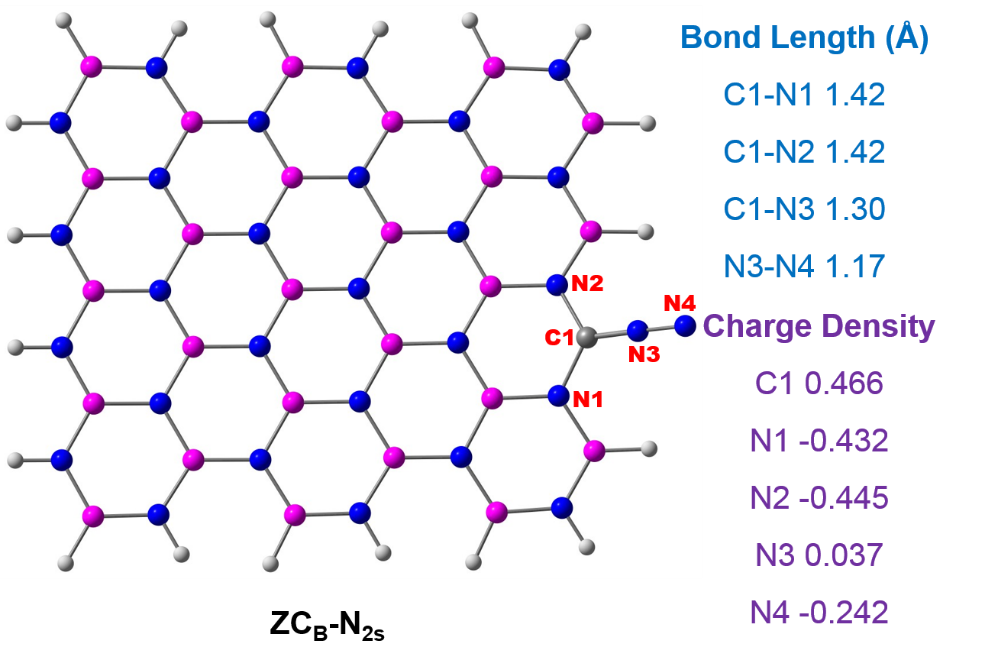


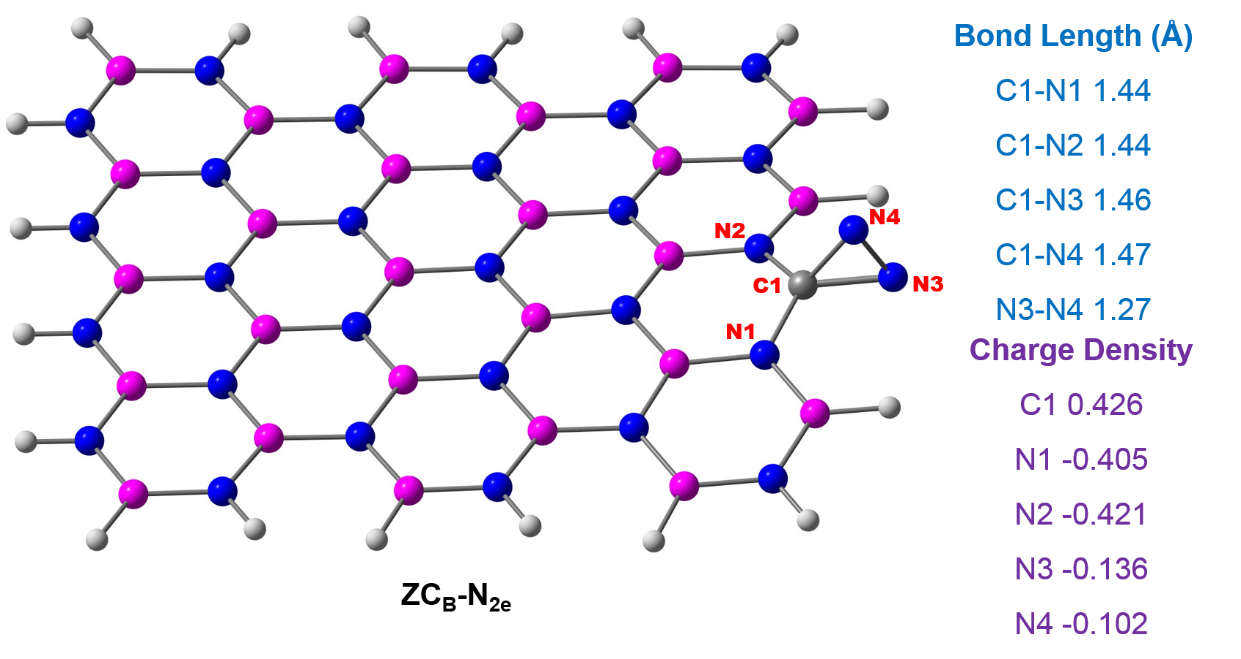


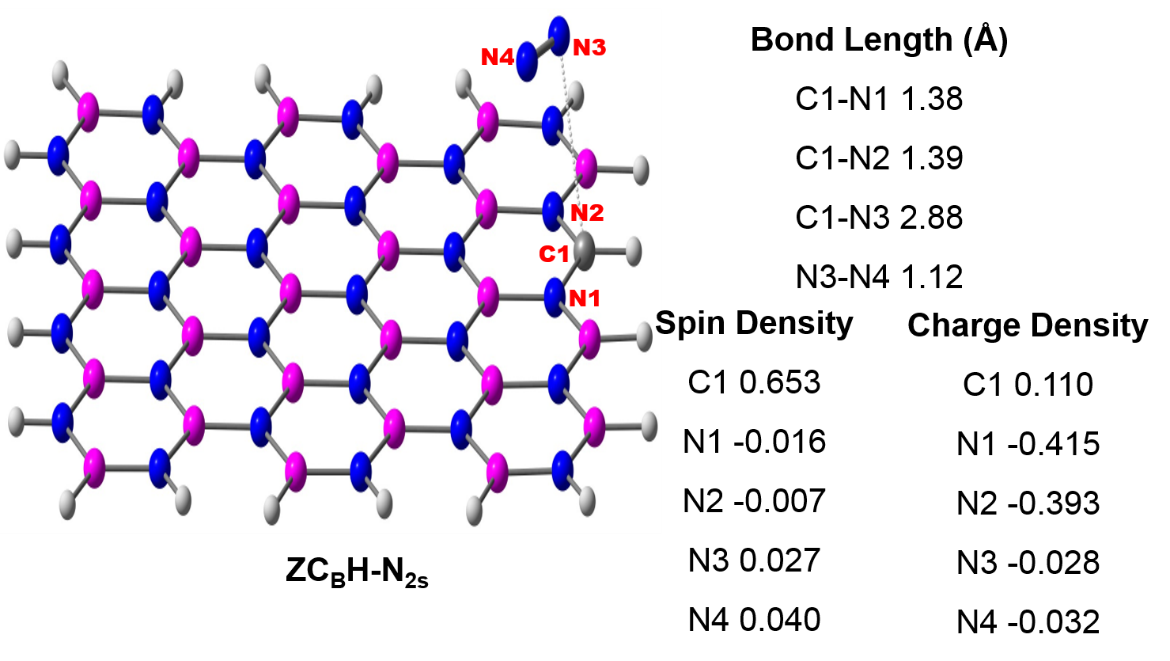





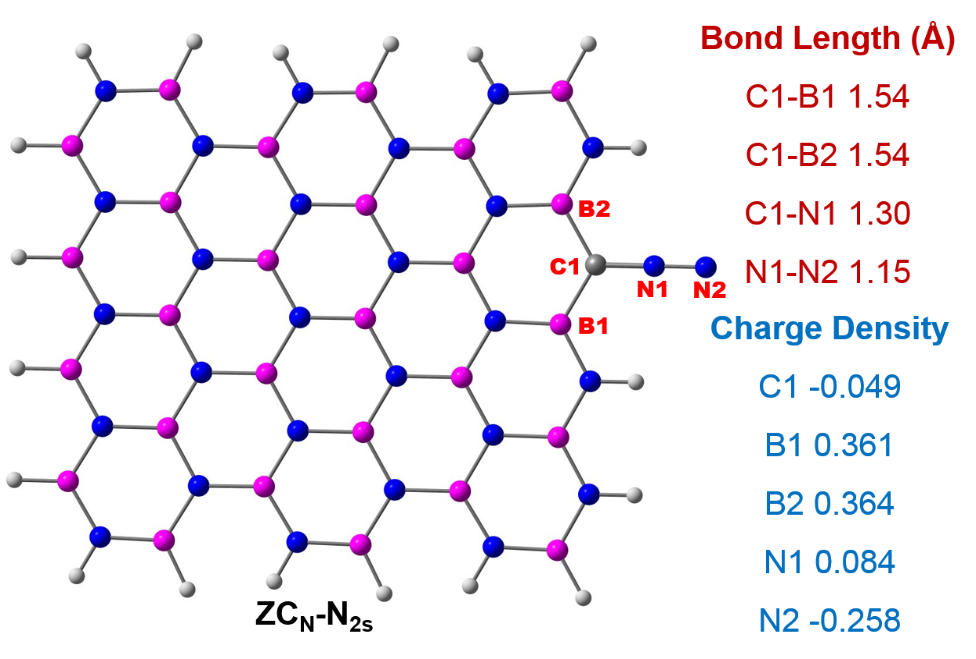


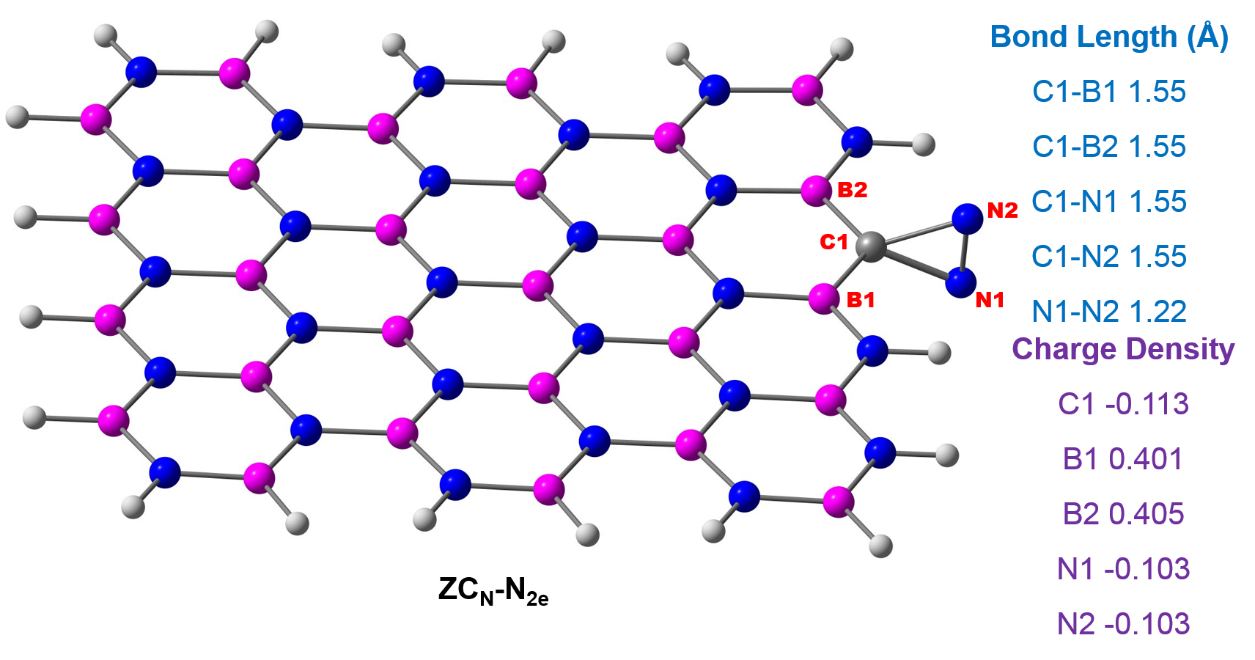


**Figure S4.** Optimized geometries of N_2_ activation by defective systems modeled by doping carbon at various zigzag edge sites calculated at PBE/6-31G(d) Level of Theory.


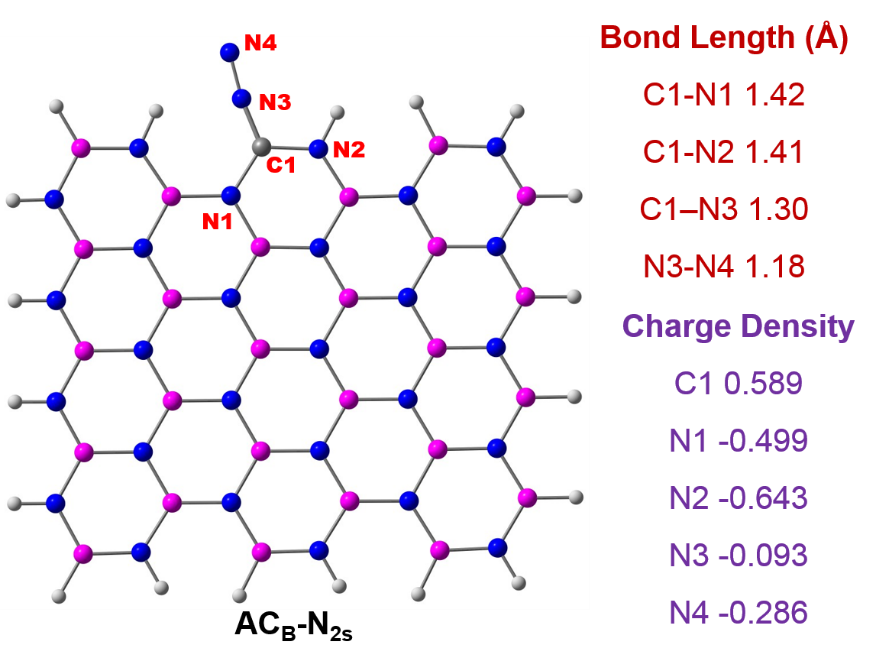


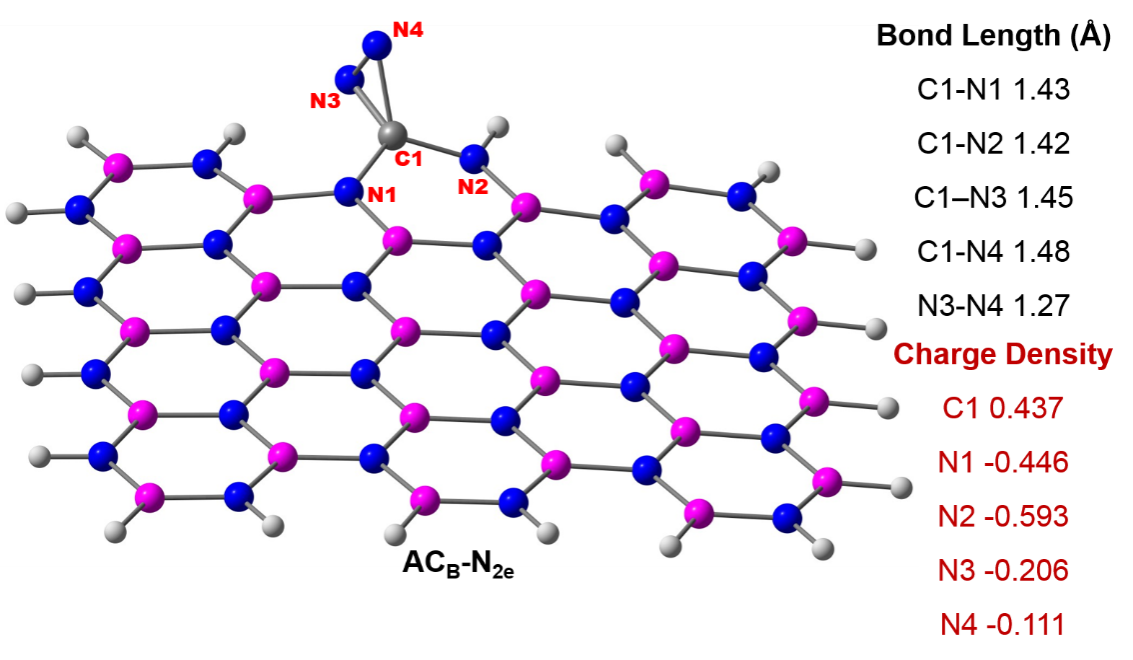


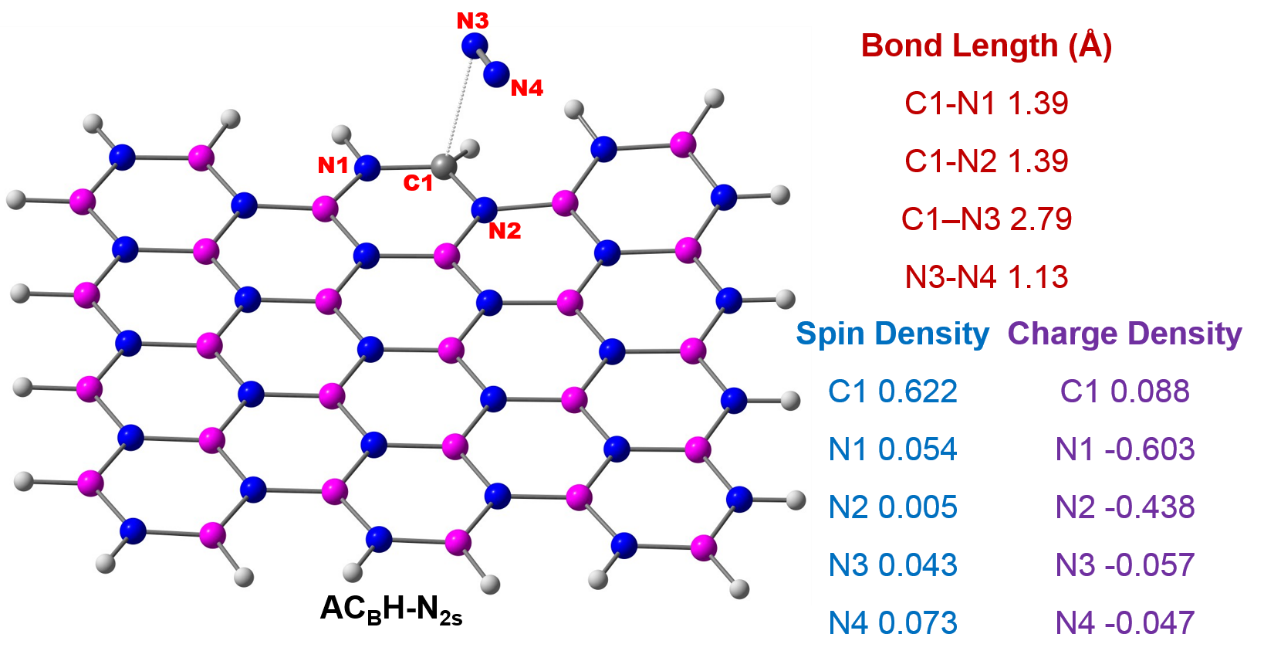


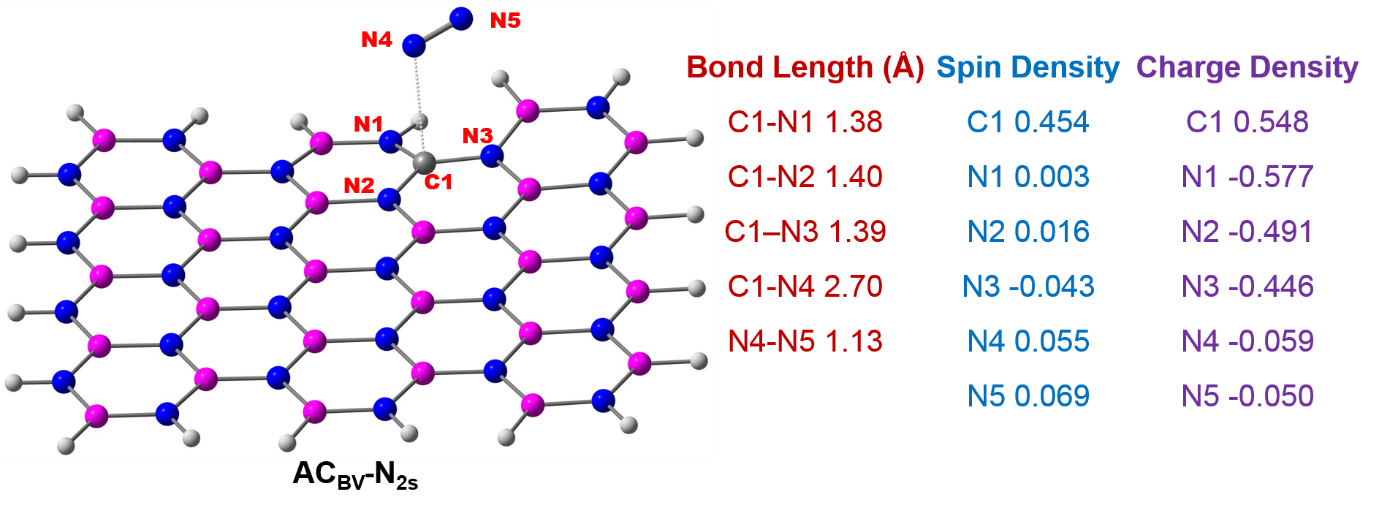


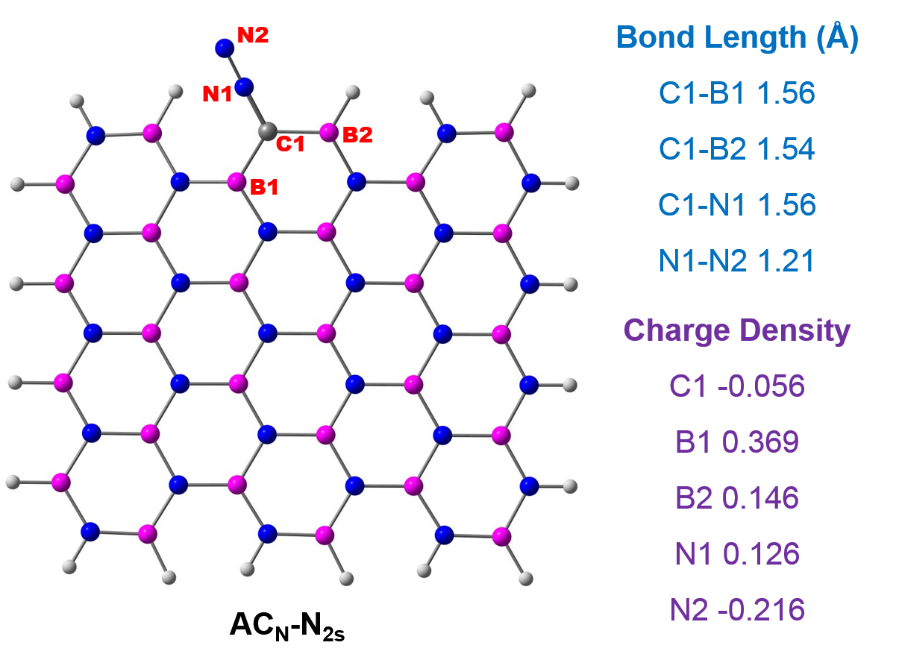


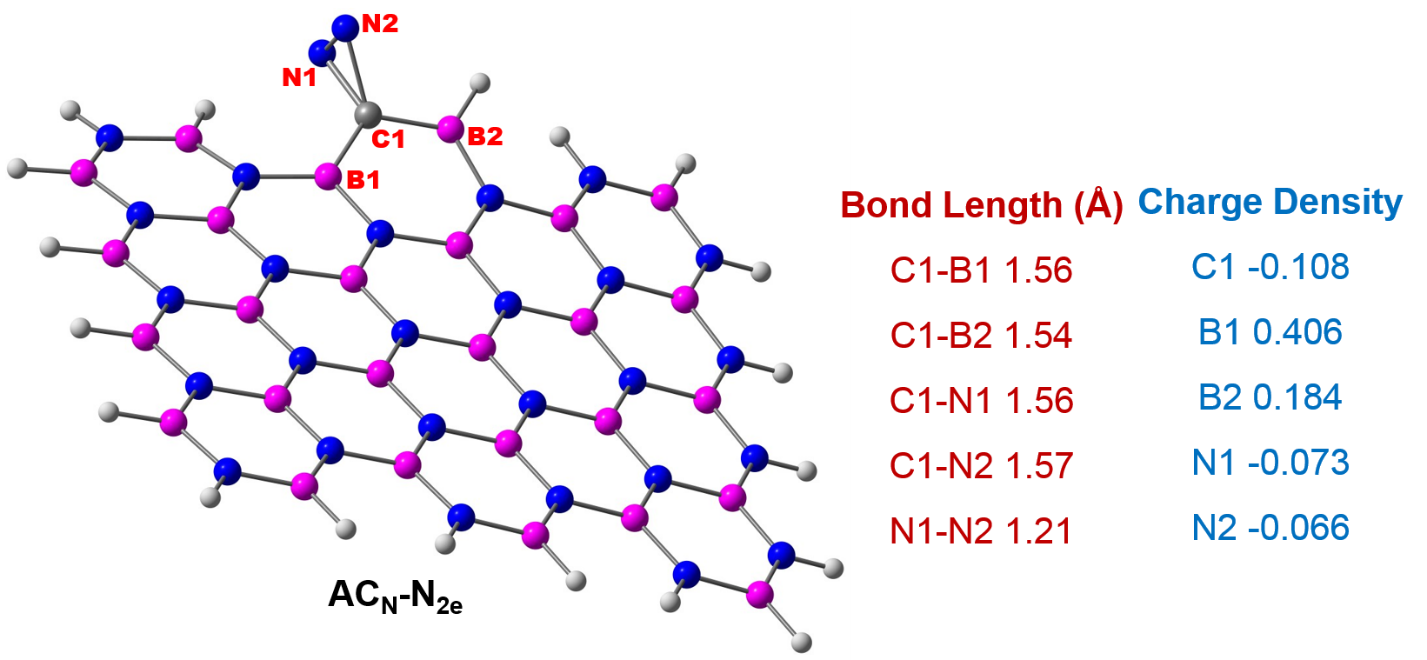


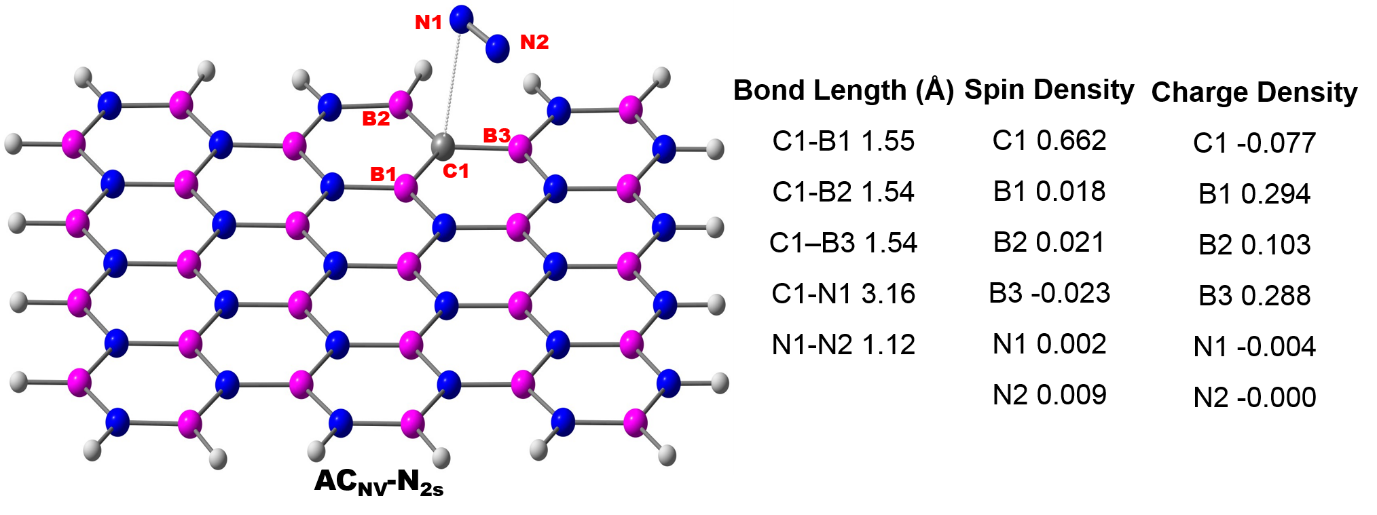


**Figure S5.** Optimized geometries of N_2_ activation by defective systems modeled by doping carbon at various armchair edge sites calculated at PBE/6-31G(d) Level of Theory.


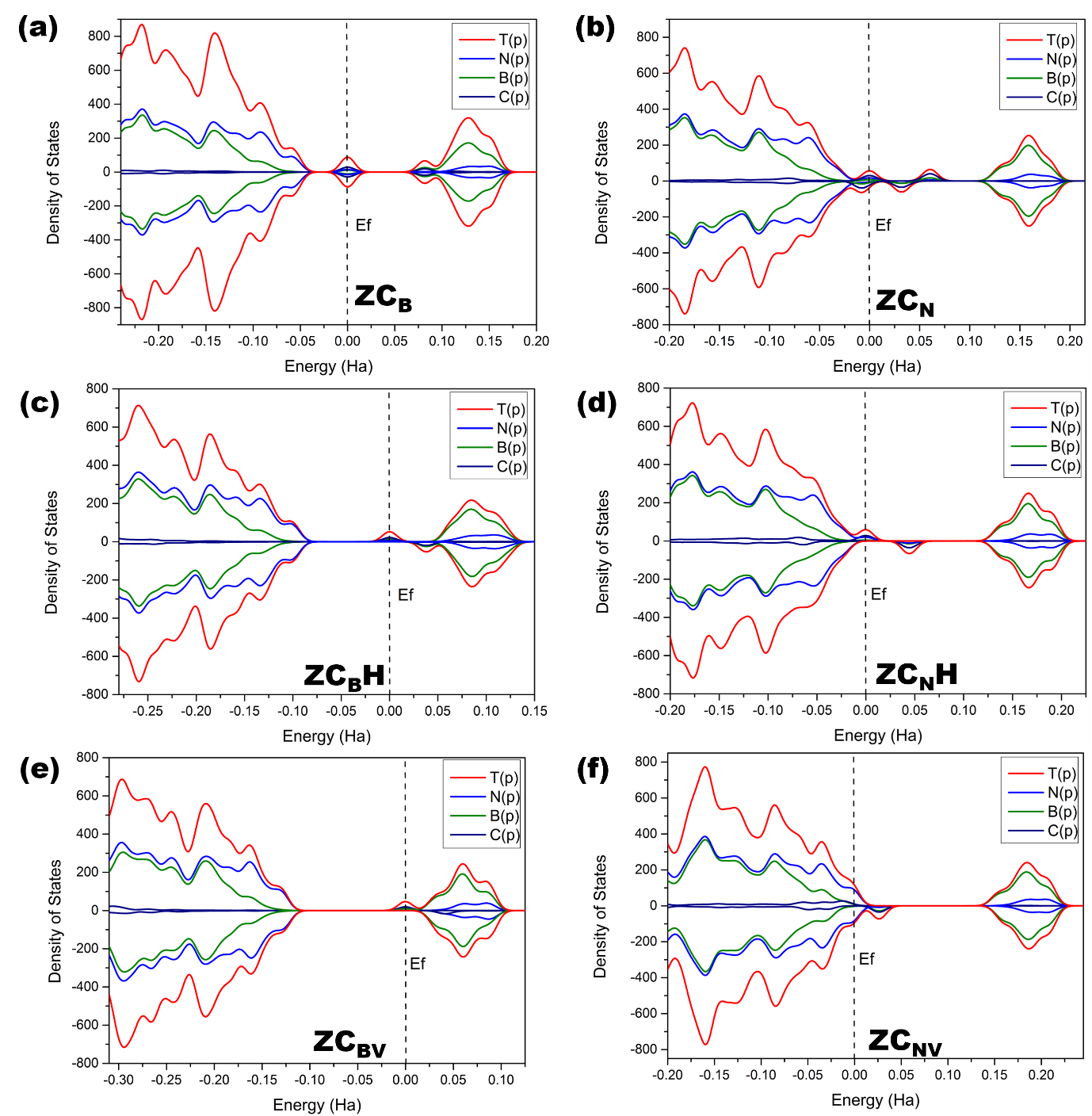


**Figure S6**. Calculated density of states (DOS) of BNS systems with carbon doping at zigzag edges. (a) ZC_B_, (b) ZC_N_, (c) ZC_B_H, (d) ZC_N_H, (e) ZC_BV_ and (f) ZC_NV_ defective BNS.


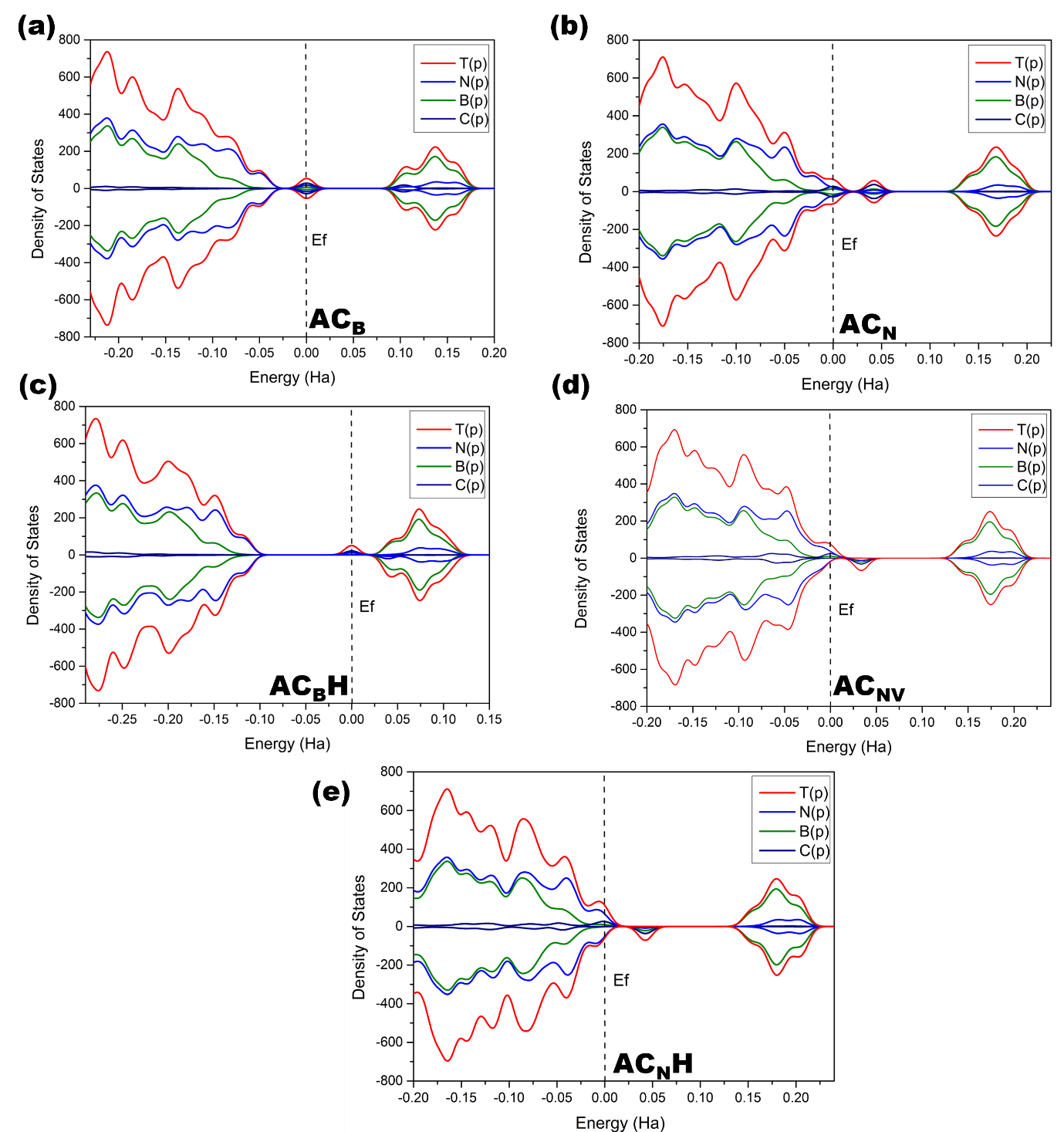


**Figure S7**. Calculated density of states (DOS) of BNS systems with carbon doping at Armchair edges. (a) AC_B_, (b) AC_N_, (c) AC_B_H, (d) AC_NV_ and (e) ZC_NH_ defective BNS.


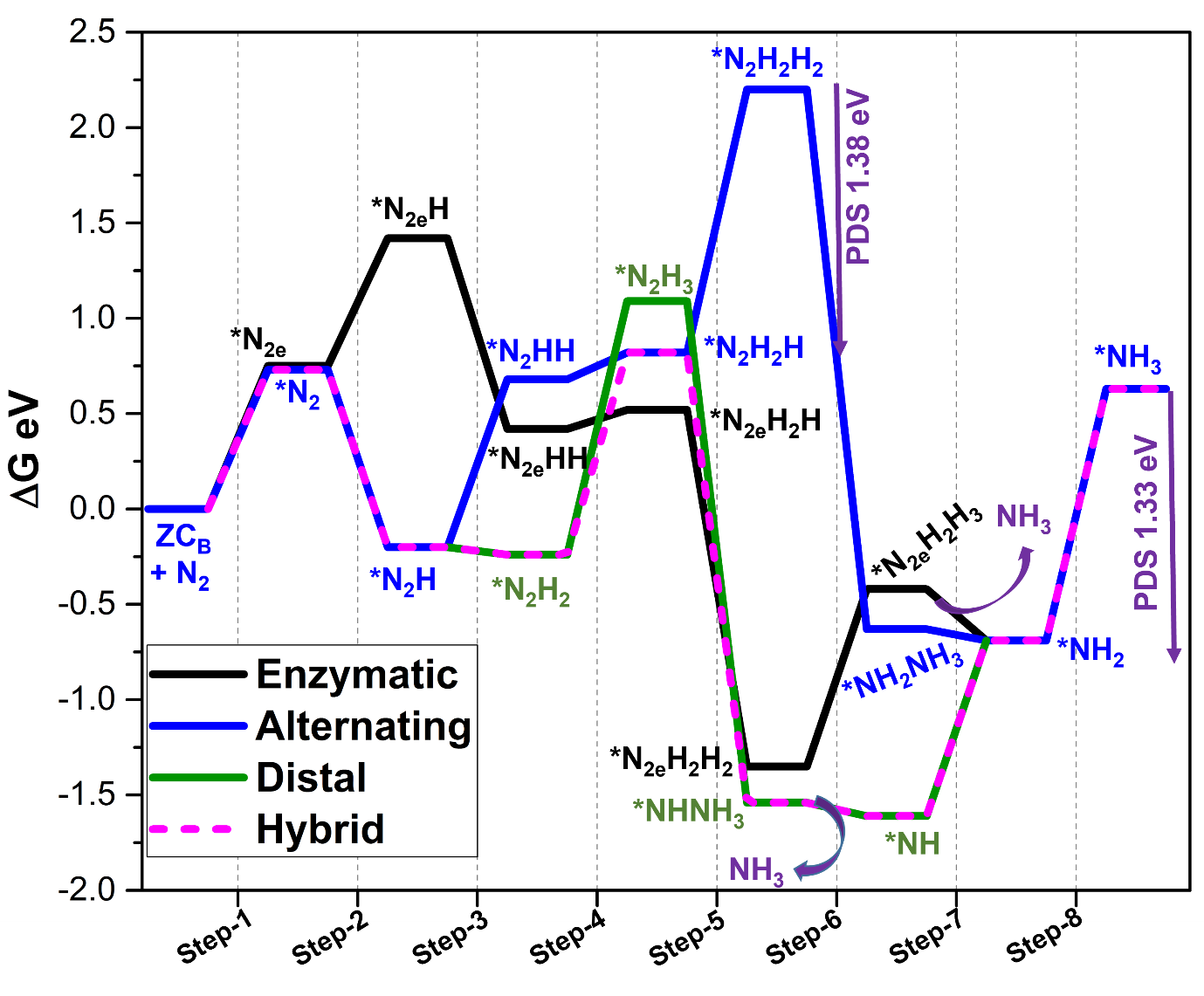


**Figure S8**. Gibbs free energy profile diagrams of possible NRR pathways catalyzed by ZC_B_ calculated at PBE/6-31G(d) level of theory.


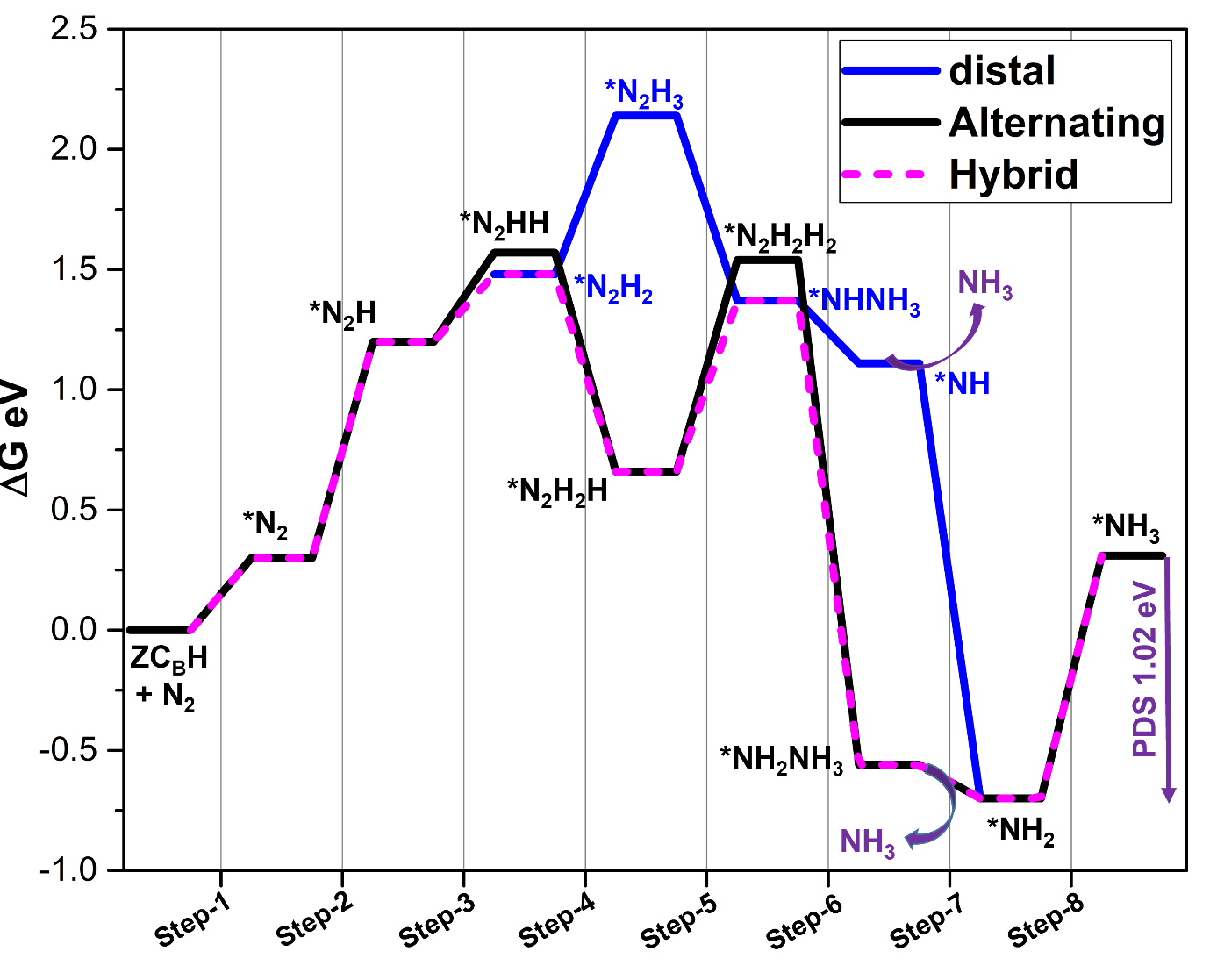


**Figure S9**. Gibbs free energy profile diagrams of possible NRR pathways catalyzed by ZC_B_H calculated at PBE/6-31G(d) level of theory.


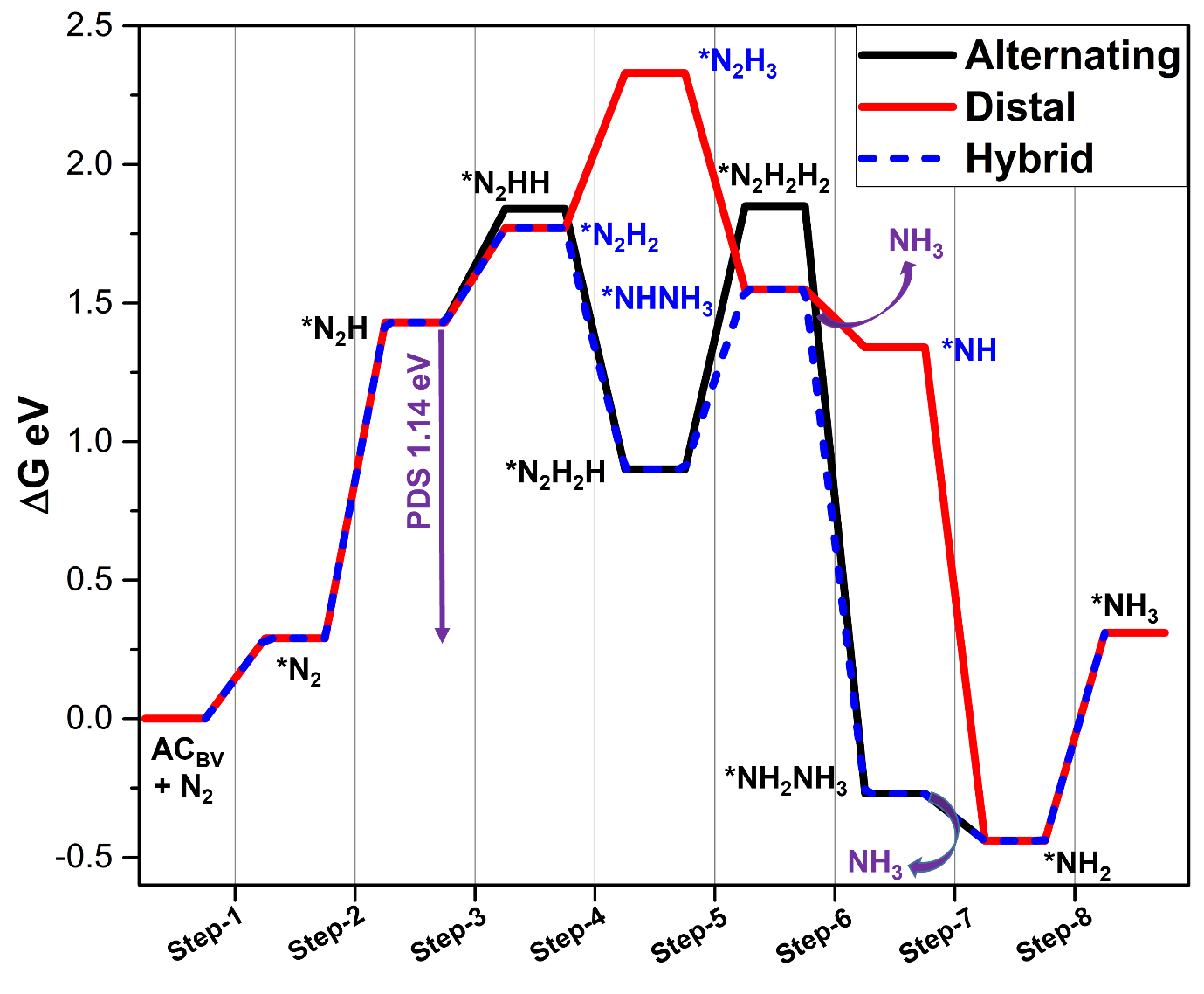


**Figure S10**. Gibbs free energy profile diagrams of possible NRR pathways catalyzed by AC_BV_ calculated at PBE/6-31G(d) level of theory.


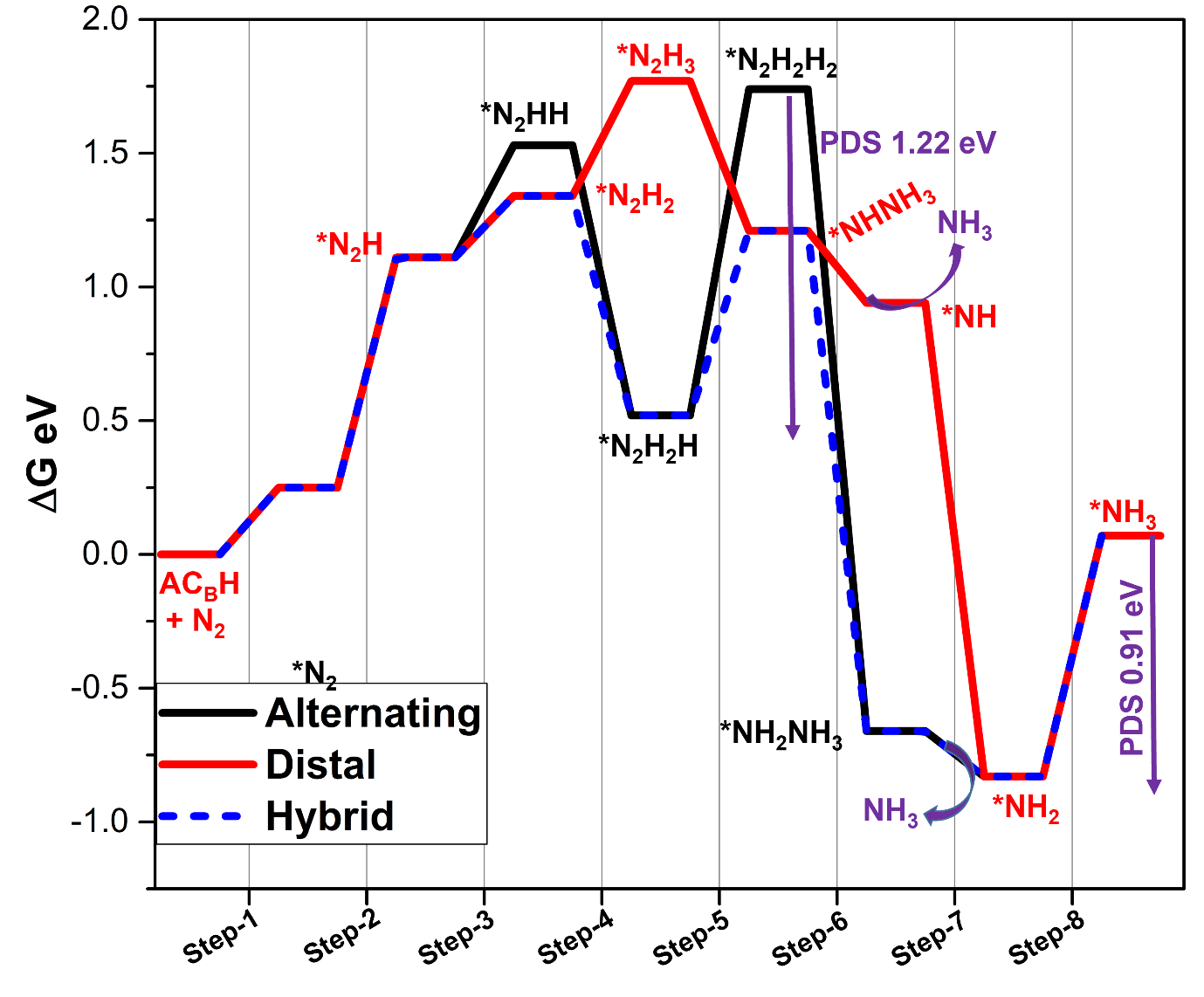


**Figure S11**. Gibbs free energy profile diagrams of possible NRR pathways catalyzed by AC_B_H calculated at PBE/6-31G(d) level of theory.


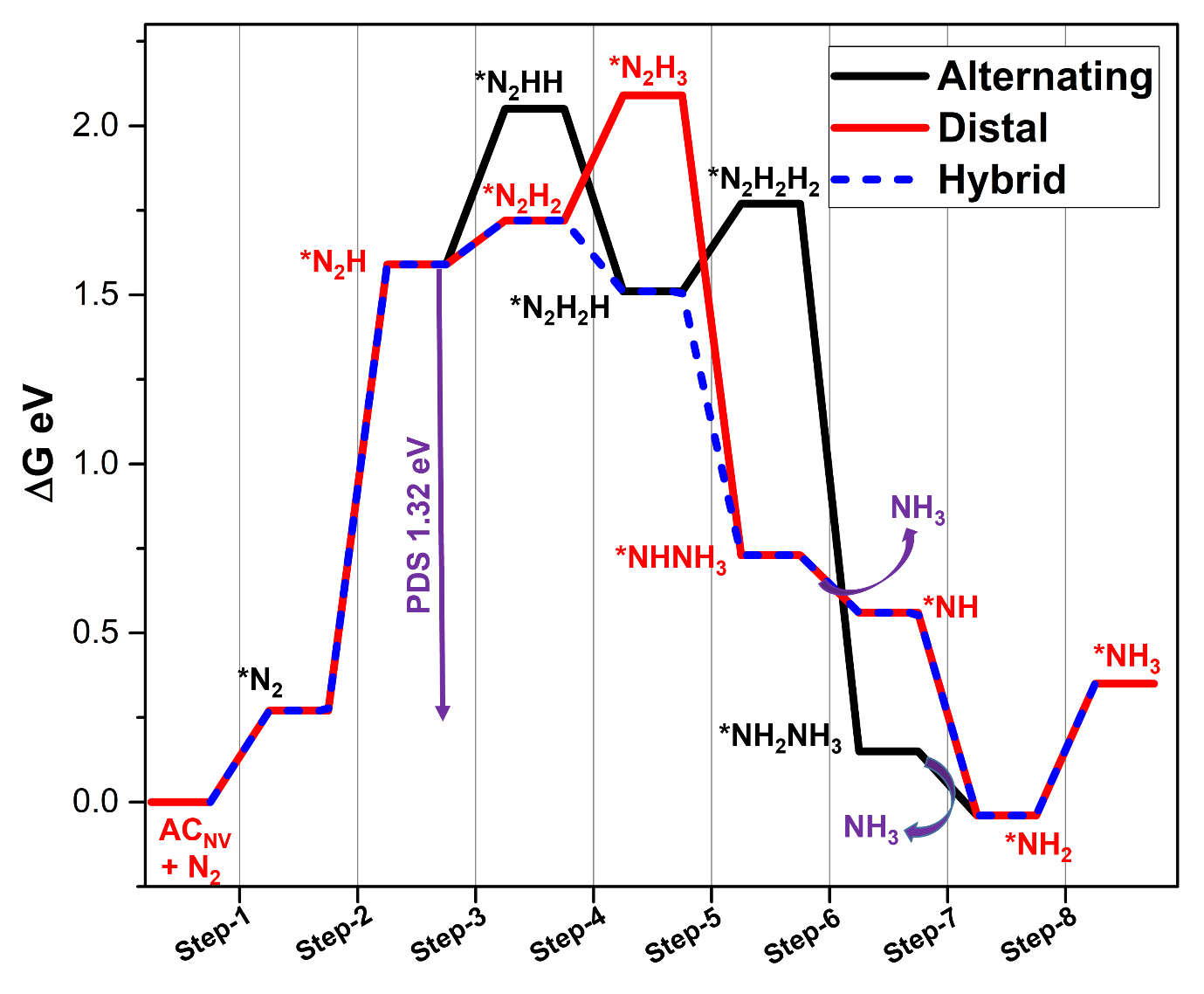


**Figure S12**. Gibbs free energy profile diagrams of possible NRR pathways catalyzed by AC_NV_ calculated at PBE/6-31G(d) level of theory.

**Table S1**. The Calculated Energetics of Elementary Steps involved in Various NRR Pathways Catalyzed by **ZC_B_** at PBE/6-31G(d) Level of Theory.

| **ZC_B_** | | | | | | | | | | | | | |
| --- | --- | --- | --- | --- | --- | --- | --- | --- | --- | --- | --- | --- | --- |
| **Elementary**  **Step** | **Distal** | | | | **Alternating** | | | **Hybrid** | | **Enzymatic** | | | |
|  | **Description** | **∆G_i_** | **∆G_R_** | **Description** | | **∆G_i_** | **∆G_R_** | **∆G_i_** | **∆G_R_** | | **Description** | **∆G_i_** | **∆G_R_** |
| **Step-1** | N_2_ activation | 0.73 | 0.73 | N_2_ activation | | **0.73** | **0.73** | **0.73** | **0.73** | | N_2_ activation | 0.75 | 0.75 |
| **Step-2** | First Protonation  (*N_2_H formation) | -0.93 | -0.20 | First Protonation  (N_2_H formation) | | **-0.93** | **-0.20** | **-0.93** | **-0.20** | | First Protonation  (N_2_H formation) | 0.67 | 1.42 |
| **Step-3** | Formation of *N_2_H_2_ | -0.05 | -0.25 | Formation of N_2_HH | | **0.87** | **0.67** | **-0.05** | **-0.25** | | Formation of N_2_HH | -1.0 | 0.42 |
| **Step-4** | Formation of *N_2_H_3_ | 1.34 | 1.09 | Formation of N_2_H_2_H  Shift from distal to alternating | | **0.15** | **0.82** | **1.06** | **0.82** | | Formation of N_2_H_2_H  Shift from distal to alternating | 0.10 | 0.52 |
| **Step-5** | Formation of the first NH_3_; Shift from alternating to distal | -2.65 | -1.56 | Formation of hydrazine intermediate | | **1.38** | **2.20** | **-2.38** | **-1.56** | | Formation of hydrazine intermediate | -1.87 | -1.35 |
| **Step-6** | NH_3_ desorption | -0.06 | -1.62 | Formation of first NH_3_ | | **-2.83** | **-0.63** | **-0.06** | **-1.62** | | Formation of first NH_3_ | 0.93 | -0.42 |
| **Step-7** | *NH_2_ formation | 0.91 | -0.71 | NH_3_ desorption | | **-0.06** | **-0.69** | **0.91** | **-0.71** | | NH_3_ desorption | -0.27 | -0.69 |
| **Step-8** | Second NH_3_ formation & PDS | 1.42 | 0.72 | Second NH_3_ formation & PDS | | **1.42** | **0.72** | **1.42** | **0.72** | | Second NH_3_ formation & PDS | 1.42 | 0.73 |

**Table S2**. The Calculated Energetics of Elementary Steps involved in Various NRR Pathways Catalyzed by **ZC_B_H** at PBE/6-31G(d) Level of Theory.

| **ZC_B_H** | | | | | | | | | | |
| --- | --- | --- | --- | --- | --- | --- | --- | --- | --- | --- |
| **Elementary**  **Step** | **Distal** | | | | **Alternating** | | | **Hybrid** | | |
|  | **Description** | **∆G_i_** | **∆G_R_** | **Description** | | **∆G_i_** | **∆G_R_** | **Description** | **∆G_i_** | **∆G_R_** |
| **Step-1** | N_2_ activation | 0.31 | 0.31 | N_2_ activation | | 0.31 | 0.31 | N_2_ Activation | 0.31 | 0.31 |
| **Step-2** | First Protonation (*N_2_H formation) | 0.89 | 1.20 | First Protonation (N_2_H formation) | | 0.89 | 1.20 | ${*N}_{2s}\to*N_{2s}H$ | 0.89 | 1.20 |
| **Step-3** | Formation of *N_2_H_2_ | 0.28 | 1.48 | Formation of N_2_HH | | 0.37 | 1.57 | ${*N}_{2s}H\to*N_{2s}H_{2}$ | 0.28 | 1.48 |
| **Step-4** | Formation of *N_2_H_3_ | 0.66 | 2.14 | Formation of N_2_H_2_H  Shift from distal to alternating | | -0.91 | 0.66 | ${*N}_{2s}H_{2}\to*N_{2s}H_{2}H$ | -0.81 | 0.66 |
| **Step-5** | Formation of the first NH_3_; Shift from alternating to distal | -0.78 | 1.37 | Formation of hydrazine intermediate | | 0.88 | 1.54 | $*N_{2}H_{2}H\to*NH+\mathrm{NH}_{3}$ | 0.71 | 1.37 |
| **Step-6** | NH_3_ desorption | -0.26 | 1.11 | Formation of first NH_3_ | | -2.11 | -0.57 | $*NH+\mathrm{NH}_{3}\to*{NH}_{2}+\mathrm{NH}_{3}$ | -1.93 | -0.57 |
| **Step-7** | *NH_2_ formation | -1.81 | -0.70 | NH_3_ desorption | | -0.14 | -0.70 | NH_3_ desorption | -0.14 | -0.70 |
| **Step-8** | Second NH_3_ formation & PDS | 1.02 | 0.32 | Second NH_3_ formation & PDS | | 1.02 | 0.32 | ${*NH}_{2}\to{*NH}_{3}$ | 1.02 | 0.32 |

**Table S3**. The Calculated Energetics of Elementary Steps involved in Various NRR Pathways Catalyzed by **ZC_BV_** at PBE/6-31G(d) Level of Theory.

| **ZC_BV_** | | | | | | | | | | | |
| --- | --- | --- | --- | --- | --- | --- | --- | --- | --- | --- | --- |
| **Elementary**  **Step** | **Distal** | | | **Alternating** | | | **Hybrid** | | | |  |
|  | **Description** | **∆G_i_** | **∆G_R_** | **Description** | **∆G_i_** | **∆G_R_** | **Description** | **∆G_i_** | **∆G_R_** |  |  |
| **Step-1** | N_2_ activation | 0.22 | 0.22 | N_2_ activation | **0.22** | **0.22** | N_2_ Activation | 0.22 | 0.22 |  |  |
| **Step-2** | First Protonation (*N_2_H formation) | 0.86 | 1.08 | First Protonation (*N_2_H formation) | **0.86** | **1.08** | ${*N}_{2s}\to*N_{2s}H$ | 0.86 | 1.08 |  |  |
| **Step-3** | Formation of *N_2_H_2_ | 0.52 | 1.60 | Formation of *N_2_HH | **0.53** | **1.61** | ${*N}_{2s}H\to*N_{2s}H_{2}$ | 0.52 | 1.60 |  |  |
| **Step-4** | Formation of *N_2_H_3_ | 0.49 | 2.09 | Formation of *N_2_H_2_H  Shift from distal to alternating | **-0.90** | **0.71** | ${*N}_{2s}H_{2}\to*N_{2s}H_{2}H$ | -0.89 | 0.71 |  |  |
| **Step-5** | Formation of the first NH_3_; Shift from alternating to distal | -0.84 | 1.25 | Formation of hydrazine intermediate | **0.23** | **0.94** | $*N_{2}H_{2}H\to*N_{2}H_{2}H_{2}$ | 0.23 | 0.94 |  |  |
| **Step-6** | NH_3_ desorption | -0.21 | 1.04 | Formation of first NH_3_ | **-1.28** | **-0.34** | $*N_{2}H_{2}H_{2}\to*{NH}_{2}+\mathrm{NH}_{3}$ | -1.28 | -0.34 |  |  |
| **Step-7** | *NH_2_ formation | -1.63 | -0.59 | NH_3_ desorption | **-0.25** | **-0.59** | NH_3_ desorption | -0.25 | -0.59 |  |  |
| **Step-8** | Second NH_3_ formation & PDS | 0.84 | 0.25 | Second NH_3_ formation & PDS | **0.84** | **0.25** | ${*NH}_{2}\to{*NH}_{3}$ | 0.84 | 0.25 |  |  |

**Table S4**. The Calculated Energetics of Elementary Steps involved in Various NRR Pathways Catalyzed by **ZC_N_** at PBE/6-31G(d) Level of Theory.

| **ZC_N_** | | | | | | | | | | | | | | |
| --- | --- | --- | --- | --- | --- | --- | --- | --- | --- | --- | --- | --- | --- | --- |
| **Elementary**  **Step** | **Distal** | | | | **Alternating** | | | **Hybrid** | | | **Enzymatic** | | |  |
|  | **Description** | **∆G_i_** | **∆G_R_** | **Description** | | **∆G_i_** | **∆G_R_** | **∆G_i_** | **∆G_R_** | **Description** | | **∆G_i_** | **∆G_R_** |  |
| **Step-1** | N_2_ activation | -2.17 | -2.17 | N_2_ activation | | -2.17 | -2.17 | **-2.17** | **-2.17** | N_2_ activation | | -0.92 | -0.92 |  |
| **Step-2** | First Protonation (*N_2_H) & PDS format | 1.13 | -1.04 | First Protonation  (N_2_H formation) | | 1.13 | -1.04 | **1.13** | **-1.04** | First Protonation  (N_2_H formation) | | 0.86 | -0.06 |  |
| **Step-3** | Formation of *N_2_H_2_ | -0.75 | -1.79 | Formation of *N_2_HH | | -0.45 | -1.49 | **-0.75** | **-1.79** | Formation of N_2_HH | | -0.90 | -0.96 |  |
| **Step-4** | Formation of *N_2_H_3_ | 1.81 | 0.02 | Formation of *N_2_H_2_H  Shift from distal to alternating | | 0.31 | -1.18 | **0.31** | **-1.18** | Formation of N_2_H_2_H  Shift from distal to alternating | | -0.22 | -1.18 |  |
| **Step-5** | Formation of the first NH_3_; Shift from alternating to distal | -2.51 | -2.49 | Formation of hydrazine intermediate | | 0.50 | -0.68 | **-2.51** | **-2.49** | Formation of hydrazine intermediate | | -1.16 | -2.34 |  |
| **Step-6** | NH_3_ desorption | -0.07 | -2.56 | Formation of first NH_3_ | | -1.49 | -2.17 | **-0.07** | **-2.56** | Formation of first NH_3_ | | 0.18 | -2.17 |  |
| **Step-7** | *NH_2_ formation | 0.03 | -2.53 | NH_3_ desorption | | -0.36 | -2.53 | **0.03** | **-2.53** | NH_3_ desorption | | -0.36 | -2.53 |  |
| **Step-8** | Second NH_3_ formation | 0.84 | -1.69 | Second NH_3_ formation & PDS | | 0.84 | -1.68 | **0.84** | **-1.69** | Second NH_3_ formation & PDS | | 0.84 | -1.68 |  |

**Table S5.**The Calculated Energetics of Elementary Steps involved in Various NRR Pathways Catalyzed by **AC_B_** at PBE/6-31G(d) Level of Theory.Blue color-hybrid pathway; Red color-PDS and Purple color-Enzymatic pathway.

| **AC_B_** | | | | | | | | | |
| --- | --- | --- | --- | --- | --- | --- | --- | --- | --- |
| **Elementary**  **Step** | **Distal** | | | **Alternating** | | | **Enzymatic** | | |
|  | **Description** | **∆G_i_** | **∆G_R_** | **Description** | **∆G_i_** | **∆G_R_** | **Description** | **∆G_i_** | **∆G_R_** |
| **Step-1** | N_2_ Activation | 1.51 | 1.51 | N_2_ Activation | 1.51 | 1.51 | N_2_ Activation | 1.33 | 1.33 |
| **Step-2** | ${*N}_{2s}\to*N_{2s}H$ | -1.03 | 0.48 | ${*N}_{2s}\to*N_{2s}H$ | -1.03 | 0.48 | ${*N}_{2e}\to*N_{2e}H$ | 0.57 | 1.89 |
| **Step-3** | ${*N}_{2s}H\to*N_{2s}H_{2}$ | -0.82 | -0.34 | ${*N}_{2s}H\to*N_{2s}\mathrm{HH}$ | 0.55 | 1.03 | ${*N}_{2e}H\to*N_{2e}\mathrm{HH}$ | -0.70 | 1.19 |
| **Step-4** | ${*N}_{2s}H_{2}\to*N+\mathrm{NH}_{3}$ | 0.60 | 0.26 | ${*N}_{2s}HH\to*N_{2s}H_{2}H$ | 0.48 | 1.51 | ${*N}_{2e}HH\to*N_{2e}H_{2}H$ | -0.31 | 0.88 |
| **Step-5** | NH_3_ desorption | -0.03 | 0.23 | ${*N}_{2s}H_{2}H\to*N_{2s}H_{2}H_{2}$ | 0.49 | 1.99 | ${*N}_{2e}H_{2}H\to*N_{2e}H_{2}H_{2}$ | -1.80 | -0.92 |
| **Step-6** | $*N\to*NH$ | -1.69 | -1.45 | $*N_{2s}H_{2}H_{2}\to{*NH}_{2}+NH_{3}$ | -1.81 | 0.18 | $*N_{2e}H_{2}H_{2}\to{*NH}_{2}+NH_{3}$ | 1.11 | 0.18 |
| **Step-7** | $*NH\to{*NH}_{2}$ | 1.45 | 0.004 | NH_2_ desorption | -0.18 | 0.004 | NH_2_ desorption | -0.18 | 0.004 |
| **Step-8** | ${*NH}_{2}\to{*NH}_{3}$ | -0.05 | -0.04 | ${*NH}_{2}\to{*NH}_{3}$ | -0.05 | -0.04 | ${*NH}_{2}\to{*NH}_{3}$ | -0.05 | -0.04 |

**Table S6**. The Calculated Energetics of Elementary Steps involved in Various NRR Pathways Catalyzed by **AC_BV_** at PBE/6-31G(d) Level of Theory.Blue color-hybrid pathway; Red color-PDS.

| **AC_BV_** | | | | | | | | | | | |
| --- | --- | --- | --- | --- | --- | --- | --- | --- | --- | --- | --- |
| **Elementary**  **Step** | **Distal** | | | | **Alternating** | | | | **Hybrid** | | |
|  | **Description** | **∆G_i_** | **∆G_R_** | **Description** | | **∆G_i_** | **∆G_R_** | **Description** | | **∆G_i_** | **∆G_R_** |
| **Step-1** | N_2_ Activation | 0.29 | 0.29 | N_2_ Activation | | 0.29 | 0.29 | N_2_ Activation | | 0.29 | 0.29 |
| **Step-2** | ${*N}_{2s}\to*N_{2s}H$ | 1.14 | 1.43 | ${*N}_{2s}\to*N_{2s}H$ | | 1.14 | 1.43 | ${*N}_{2s}\to*N_{2s}H$ | | 1.14 | 1.43 |
| **Step-3** | ${*N}_{2s}H\to*N_{2s}H_{2}$ | 0.34 | 1.77 | ${*N}_{2s}H\to*N_{2s}\mathrm{HH}$ | | 0.41 | 1.84 | ${*N}_{2s}H\to*N_{2s}H_{2}$ | | 0.34 | 1.77 |
| **Step-4** | ${*N}_{2s}H_{2}\to*N_{2}H_{3}$ | 0.57 | 2.34 | ${*N}_{2s}HH\to*N_{2s}H_{2}H$ | | -0.94 | 0.90 | ${*N}_{2s}H_{2}\to*N_{2s}H_{2}H$ | | -0.87 | 0.90 |
| **Step-5** | $*N_{2}H_{3}\to*NH+\mathrm{NH}_{3}$ | -0.79 | 1.55 | ${*N}_{2s}H_{2}H\to*N_{2s}H_{2}H_{2}$ | | 0.95 | 1.85 | $*N_{2}H_{2}H\to*NH+\mathrm{NH}_{3}$ | | 0.65 | 1.55 |
| **Step-6** | NH_3_ desorption | -0.21 | 1.34 | $*N_{2s}H_{2}H_{2}\to{*NH}_{2}+NH_{3}$ | | -2.12 | -0.27 | $*NH+\mathrm{NH}_{3}\to*{NH}_{2}+\mathrm{NH}_{3}$ | | -1.82 | -0.27 |
| **Step-7** | $*NH\to{*NH}_{2}$ | -1.79 | -0.45 | NH_2_ desorption | | -0.17 | -0.45 | NH_2_ desorption | | -0.17 | -0.45 |
| **Step-8** | ${*NH}_{2}\to{*NH}_{3}$ | 0.76 | 0.32 | ${*NH}_{2}\to{*NH}_{3}$ | | 0.76 | 0.32 | ${*NH}_{2}\to{*NH}_{3}$ | | 0.76 | 0.32 |

**Table S7**. The Calculated Energetics of Elementary Steps involved in Various NRR Pathways Catalyzed by **AC_B_H** at PBE/6-31G(d) Level of Theory.Blue color-hybrid pathway; Red color-PDS.

| **AC_B_H** | | | | | | | | | |
| --- | --- | --- | --- | --- | --- | --- | --- | --- | --- |
| **Elementary**  **Step** | **Distal** | | | **Alternating** | | | **Hybrid** | | |
|  | **Description** | **∆G_i_** | **∆G_R_** | **Description** | **∆G_i_** | **∆G_R_** | **Description** | **∆G_i_** | **∆G_R_** |
| **Step-1** | N_2_ Activation | 0.25 | 0.25 | N_2_ Activation | 0.25 | 0.25 | N_2_ Activation | 0.25 | 0.25 |
| **Step-2** | ${*N}_{2s}\to*N_{2s}H$ | 0.86 | 1.11 | ${*N}_{2s}\to*N_{2s}H$ | 0.86 | 1.11 | ${*N}_{2s}\to*N_{2s}H$ | 0.86 | 1.11 |
| **Step-3** | ${*N}_{2s}H\to*N_{2s}H_{2}$ | 0.22 | 1.34 | ${*N}_{2s}H\to*N_{2s}\mathrm{HH}$ | 0.41 | 1.53 | ${*N}_{2s}H\to*N_{2s}H_{2}$ | 0.22 | 1.34 |
| **Step-4** | ${*N}_{2s}H_{2}\to*N_{2}H_{3}$ | 0.43 | 1.77 | ${*N}_{2s}HH\to*N_{2s}H_{2}H$ | -1.01 | 0.52 | ${*N}_{2s}H_{2}\to*N_{2s}H_{2}H$ | -0.82 | 0.52 |
| **Step-5** | $*N_{2}H_{3}\to*NH+\mathrm{NH}_{3}$ | -0.56 | 1.21 | ${*N}_{2s}H_{2}H\to*N_{2s}H_{2}H_{2}$ | 1.22 | 1.74 | ${*N}_{2s}H_{2}H\to*NH+\mathrm{NH}_{3}$ | 0.69 | 1.21 |
| **Step-6** | NH_3_ desorption | -0.26 | 0.95 | $*N_{2s}H_{2}H_{2}\to{*NH}_{2}+NH_{3}$ | -2.40 | -0.66 | $*NH+\mathrm{NH}_{3}\to{*NH}_{2}+NH_{3}$ | -1.85 | -0.66 |
| **Step-7** | $*NH\to{*NH}_{2}$ | -1.78 | -0.84 | NH_2_ desorption | -0.17 | -0.84 | NH_3_ desorption | -0.17 | -0.84 |
| **Step-8** | ${*NH}_{2}\to{*NH}_{3}$ | 0.91 | 0.07 | ${*NH}_{2}\to{*NH}_{3}$ | 0.91 | 0.07 | ${*NH}_{2}\to{*NH}_{3}$ | 0.91 | 0.07 |

**Table S8**. The Calculated Energetics of Elementary Steps involved in Various NRR Pathways Catalyzed by **AC_N_**at PBE/6-31G(d) Level of Theory.Blue color-hybrid pathway; Red color-PDS and Purple color-Enzymatic pathway.

| **AC_N_** | | | | | | | | | | | | | |
| --- | --- | --- | --- | --- | --- | --- | --- | --- | --- | --- | --- | --- | --- |
| **Elementary**  **Step** | **Distal** | | | **Alternating** | | | **Hybrid** | | | | **Enzymatic** | | |
|  | **Description** | **∆G_i_** | **∆G_R_** | **Description** | **∆G_i_** | **∆G_R_** | **Description** | **∆G_i_** | **∆G_R_** | **Description** | | **∆G_i_** | **∆G_R_** |
| **Step-1** | N_2_ Activation | -1.84 | -1.84 | N_2_ Activation | -1.84 | -1.84 | N_2_ Activation | -1.84 | -1.84 | N_2_ Activation | | -0.39 | -0.39 |
| **Step-2** | ${*N}_{2s}\to*N_{2s}H$ | 1.46 | -0.38 | ${*N}_{2s}\to*N_{2s}H$ | 1.46 | -0.38 | ${*N}_{2s}\to*N_{2s}H$ | 1.46 | -0.38 | ${*N}_{2e}\to*N_{2e}H$ | | 0.88 | 0.49 |
| **Step-3** | ${*N}_{2s}H\to*N_{2s}H_{2}$ | -1.05 | -1.43 | ${*N}_{2s}H\to*N_{2s}\mathrm{HH}$ | -0.85 | -1.23 | ${*N}_{2s}H\to*N_{2s}H_{2}$ | -1.05 | -1.43 | ${*N}_{2e}H\to*N_{2e}\mathrm{HH}$ | | -0.75 | -0.26 |
| **Step-4** | ${*N}_{2s}H_{2}\to*N_{2}H_{3}$ | 1.43 | 0.00 | ${*N}_{2s}HH\to*N_{2s}H_{2}H$ | 0.16 | -1.07 | ${*N}_{2s}H_{2}\to*N_{2s}H_{2}H$ | 0.36 | -1.07 | ${*N}_{2e}HH\to*N_{2e}H_{2}H$ | | -0.67 | -0.94 |
| **Step-5** | $*N_{2}H_{3}\to*NH+\mathrm{NH}_{3}$ | -1.72 | -1.72 | ${*N}_{2s}H_{2}H\to*N_{2s}H_{2}H_{2}$ | 0.26 | -0.80 | ${*N}_{2s}H_{2}H\to*NH+\mathrm{NH}_{3}$ | -0.65 | -1.72 | ${*N}_{2e}H_{2}H\to*N_{2e}H_{2}H_{2}$ | | 0.12 | -0.81 |
| **Step-6** | NH_3_ desorption | -0.24 | -1.96 | $*N_{2s}H_{2}H_{2}\to{*NH}_{2}+NH_{3}$ | -1.49 | -2.30 | $*NH+\mathrm{NH}_{3}\to{*NH}_{2}+NH_{3}$ | -0.57 | -2.30 | $*N_{2e}H_{2}H_{2}\to{*NH}_{2}+NH_{3}$ | | -1.49 | -2.30 |
| **Step-7** | $*NH\to{*NH}_{2}$ | -0.42 | -2.39 | NH_2_ desorption | -0.09 | -2.39 | NH_3_ desorption | -0.09 | -2.39 | NH_2_ desorption | | -0.09 | -2.39 |
| **Step-8** | ${*NH}_{2}\to{*NH}_{3}$ | 0.44 | -1.95 | ${*NH}_{2}\to{*NH}_{3}$ | 0.44 | -1.95 | ${*NH}_{2}\to{*NH}_{3}$ | 0.44 | -1.95 | ${*NH}_{2}\to{*NH}_{3}$ | | 0.44 | -1.95 |

**Table S9**. The Calculated Energetics of Elementary Steps involved in Various NRR Pathways Catalyzed by **AC_NV_** at PBE/6-31G(d) Level of Theory. Blue color-hybrid pathway; Red color-PDS.

| **AC_NV_** | | | | | | | | | | |
| --- | --- | --- | --- | --- | --- | --- | --- | --- | --- | --- |
| **Elementary**  **Step** | **Distal** | | | **Alternating** | | | **Hybrid** | | | |
|  | **Description** | **∆G_i_** | **∆G_R_** | **Description** | **∆G_i_** | **∆G_R_** | **Description** | **∆G_i_** | **∆G_R_** |  |
| **Step-1** | N_2_ Activation | 0.27 | 0.27 | N_2_ Activation | 0.27 | 0.27 | N_2_ Activation | 0.27 | 0.27 |  |
| **Step-2** | ${*N}_{2s}\to*N_{2s}H$ | 1.32 | 1.59 | ${*N}_{2s}\to*N_{2s}H$ | 1.32 | 1.59 | ${*N}_{2s}\to*N_{2s}H$ | 1.32 | 1.59 |  |
| **Step-3** | ${*N}_{2s}H\to*N_{2s}H_{2}$ | 0.13 | 1.72 | ${*N}_{2s}H\to*N_{2s}\mathrm{HH}$ | 0.46 | 2.06 | ${*N}_{2s}H\to*N_{2s}H_{2}$ | 0.13 | 1.72 |  |
| **Step-4** | ${*N}_{2s}H_{2}\to*N_{2}H_{3}$ | 0.37 | 2.09 | ${*N}_{2s}HH\to*N_{2s}H_{2}H$ | -0.55 | 1.51 | ${*N}_{2s}H_{2}\to*N_{2s}H_{2}H$ | -0.21 | 1.51 |  |
| **Step-5** | $*N_{2}H_{3}\to*NH+\mathrm{NH}_{3}$ | -1.36 | 0.73 | ${*N}_{2s}H_{2}H\to*N_{2s}H_{2}H_{2}$ | 0.27 | 1.78 | ${*N}_{2s}H_{2}H\to*NH+\mathrm{NH}_{3}$ | -0.78 | 0.73 |  |
| **Step-6** | NH_3_ desorption | -0.17 | 0.56 | $*N_{2s}H_{2}H_{2}\to{*NH}_{2}+NH_{3}$ | -1.62 | 0.16 | $**NH+\mathrm{NH}_{3}\to{*NH}_{2}+NH_{3}$ | -0.57 | 0.16 |  |
| **Step-7** | $*NH\to{*NH}_{2}$ | -0.61 | -0.05 | NH_2_ desorption | -0.21 | -0.05 | NH_3_ desorption | -0.21 | -0.05 |  |
| **Step-8** | ${*NH}_{2}\to{*NH}_{3}$ | 0.40 | 0.35 | ${*NH}_{2}\to{*NH}_{3}$ | 0.40 | 0.35 | ${*NH}_{2}\to{*NH}_{3}$ | 0.40 | 0.35 |  |
